# When EV Dose Is Not What It Seems: Quantitative Mismatch Between Particle Number‐ and Protein‐Based Dosing

**DOI:** 10.1002/jex2.70188

**Published:** 2026-09-13

**Authors:** Tímea Böröczky, Mária Harmati, Edina Gyukity‐Sebestyén, Tamás F. Polgár, Csenge Feinek, Emma Balog, Antal Martinecz, Krisztina Buzás, Gabriella Dobra, Mátyás Bukva

**Affiliations:** ^1^ Institute of Biochemistry Biological Research Centre HUN‐REN Szeged Hungary; ^2^ Department of Immunology University of Szeged Szeged Hungary; ^3^ Doctoral School of Experimental and Preventive Medicine University of Szeged Szeged Hungary; ^4^ Transmission Electron Microscope Laboratory, Core Facility HUN‐REN Biological Research Centre Szeged Hungary; ^5^ HUN‐REN‐SZTE Neuroscience Research Group, Hungarian Research Network University of Szeged Szeged Hungary; ^6^ Certara Radnor Pennsylvania USA

**Keywords:** extracellular vesicles (EVs), protein dilution effect, protein dose, quantification, tumour, vesicular protein

## Abstract

Extracellular vesicles (EVs) are widely studied as mediators of intercellular communication, yet their quantitative characterization remains inconsistent. In this study, we investigated how vesicle release rate, cellular proliferation, and tumour origin jointly shape EV‐associated protein output. Using a panel of 19 human, murine, canine and primate cancer‐ and non‐cancer cell lines cultured under standardized conditions, we isolated small extracellular vesicle‐enriched fractions and quantified particle number, EV‐associated protein content, cell number and proliferation rate and integrated these parameters into three complementary, readily computable metrics: protein content per particle (PP; pg/particle), Vesicle Release Characteristics (VRC; particles/cell/24 h), and Protein Release Characteristics (PRC; pg/cell/24 h). Cancer cells showed significantly higher estimated EV release per cell than non‐cancer cells (∼3.6‐fold higher VRC), while their EV‐enriched isolates exhibited a substantially lower protein‐to‐particle ratio (∼4.3‐fold lower PP). Lower PP was independently associated with both cancer origin and proliferation rate and was not explained by differences in vesicle size. Despite the elevated vesicle output, cancer‐derived cells exported less total vesicular protein per cell and time unit (∼2.1‐fold lower PRC), and the relationship between vesicle output and total protein output itself differed between non‐cancer and cancer cells. These findings demonstrate that particle number and protein content describe distinct and non‐equivalent aspects of EV output. We therefore recommend integrated, multidimensional reporting (VRC, PRC, and the protein/particle descriptor PP — or vesicles per pg protein) to enable biologically meaningful comparison and dosing of EV preparations.

## Graphical abstract

Created with BioRender.com.

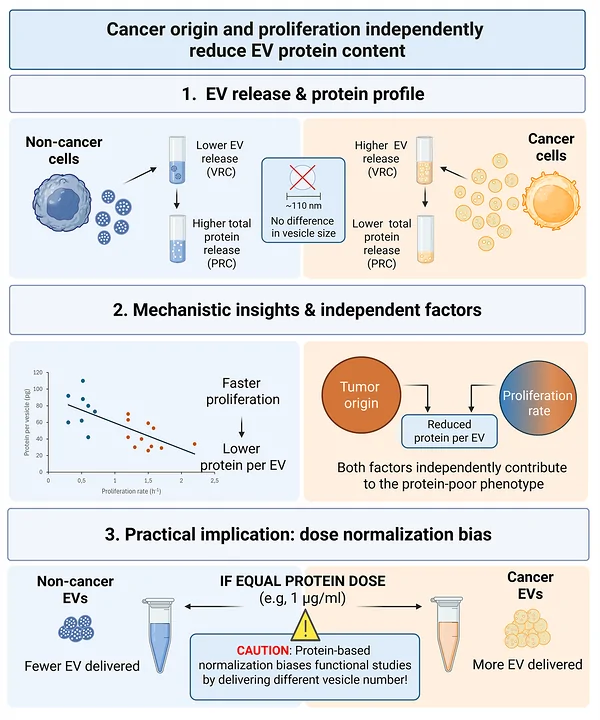

**Abbreviations**
2D: two‐dimensional
3D: three‐dimensional
APC: allophycocyanin
BCA: bicinchoninic acid
dUC: differential ultracentrifugation
EV: extracellular vesicle
EV‐TRACK: Transparent Reporting and Centralizing Knowledge in Extracellular Vesicle Research
FBS: fetal bovine serum
FC: flow cytometry
IQR: interquartile range
MACSPlex: MACSPlex Exosome Kit
MISEV: Minimal Information for Studies of Extracellular Vesicles
NTA: nanoparticle tracking analysis
PBS: phosphate‐buffered saline
PP: Protein/particle
PRC: Protein Release Characteristics
RT: room temperature
sEV: small extracellular vesicle
TEM: transmission electron microscopy
VRC: Vesicle Release Characteristics

## 1 Introduction

Extracellular vesicles (EVs) are small, membrane‐enclosed particles that are released by virtually all cell types into their extracellular environment. They carry molecular cargo, including proteins, lipids and nucleic acids and play key roles in intercellular communication, including modulation of the tumour microenvironment, regulation of immune responses and contribution to metastatic progression (Costa‐Silva et al. 2015; Hoshino et al. 2015; Kalluri and McAndrews 2023; Lopez et al. 2023; Peinado 2012; Poggio et al. 2019; Semeradtova et al. 2025).

Nowadays, EVs are widely investigated as a rich reservoir of biomarkers and as potential therapeutic tools. Consequently, beyond characterization of EV cargo, quantitative description of EV release has become increasingly important. In line with this need, several advanced single‐vesicle technologies have been developed (e.g. high‐sensitivity flow cytometry (Kobayashi et al. 2024), single‐cell secretion analysis (Pei et al. 2025), but they remain limited to specialized settings, and most studies still rely on bulk particle/protein measurements.

Accordingly, in most experimental and clinical studies EV abundance continues to be reported using particle‐based metrics, typically obtained by nanoparticle tracking analysis (NTA) or related methods, normalized to volume (particles/mL), cell number (particles/cell), and/or time (e.g., particles/cell/day) (Casajuana Ester and Day 2023; Figueroa‐Valdés et al. 2021; Geng et al. 2024; Kim et al. 2021; Ma et al. 2025).

In parallel, many studies standardize EV quantity based on total vesicular protein content, relying on the implicit assumption that a given amount of EV‐associated protein represents a proportional number of vesicles. As a result, protein mass is frequently used as a proxy for EV abundance, and dose definition or comparison is based on protein‐based normalization (e.g., µg protein or pg protein/cell) rather than particle number (Gupta et al. 2021). Both functional cancer EV studies (Tabak et al. 2018, Zhang et al. 2019) and targeted methodological reviews (Gupta et al. 2021) emphasize the substantial heterogeneity of EV dose‐normalization strategies (particle‐based versus protein‐based) across the literature.

The common, often unstated assumption underlying these practices is that a specific amount of protein corresponds to a specific number of EVs (and, conversely, that a specific EV count equates to a specific protein mass), independent of cellular origin. However, multiple observations challenge this assumption. In some systems, increased EV secretion is accompanied by reduced protein content per vesicle. Bioreactor studies, for example, have shown that endothelial or mesenchymal stem cells can increase EV yield by tens to more than a hundredfold while the average protein content per vesicle drops markedly, a phenomenon explicitly described as a ‘protein dilution effect’ (Kronstadt et al. 2023; Patel et al. 2019). Similar patterns have been reported outside bioreactor settings; for instance, neutral sphingomyelinase inhibition increases EV number while reducing protein per vesicle (Menck 2017).

These results have direct methodological implications. If EVs derived from different cellular origins, which display distinct EV release dynamics, are administered based on equal protein mass, then the actual number of vesicles delivered may differ substantially, which can bias comparisons. In contrast, fixing EV dose by particle number can likewise be misleading because differences in cargo loading per vesicle lead to unequal total protein (and cargo) delivery (Gupta et al. 2021; Huang et al. 2025; Lopez et al. 2023).

Taken together, neither particle‐based nor protein‐based quantification on its own adequately reflects EV output. Particle counts do not inform on protein content per vesicle or on the amount of material exported per cell and time unit, while bulk protein measurements cannot distinguish between scenarios involving many protein‐poor EVs versus few protein‐rich EVs. In line with this, the MISEV2023 guidelines explicitly recommend multidimensional reporting, including particle number, EV‐associated protein content, and detailed culture parameters such as cell density, incubation time, and proliferation status. They also highlight the need to avoid overreliance on any single normalization strategy (Welsh et al. 2024).

Nevertheless, only a few studies describe, within a unified framework, (i) the intensity of EV release, (ii) protein content per vesicle, and (iii) vesicular protein export normalized to cell number and time. This gap is particularly relevant in tumour biology, where numerous studies indicate that cancer‐derived EVs differ functionally and proteomically from non‐cancer EVs and, most importantly, are often released in higher amounts (Chen et al. 2022; Choi et al. 2012; Goudsmit et al. 2021; Guerreiro et al. 2020; Hurwitz et al. 2016; Priya et al. 2022; Servage et al. 2020).

If the ‘protein dilution effect’ is broadly applicable and also operates in cancer and non‐cancer cell lines beyond specialized bioreactor or perturbation settings, then current dosing practices may systematically distort functional comparisons between cancer‐derived and non‐cancer‐derived EVs. In other words, simply switching the dose normalization axis can change which EV population appears more potent, yet this possibility is rarely addressed explicitly in the EV literature.

Accordingly, we set out to test whether increased EV release is associated with reduced protein content per vesicle, and how this relationship depends on cancer origin. Given that altered growth dynamics are a fundamental characteristic of cancer cells, we further incorporated proliferation rate as a quantitative descriptor of cellular physiological state to evaluate how growth rate influences EV secretion and associated protein output.

Based on these considerations, we analyzed EV release and vesicular protein content across 19cell lines, including cancer‐derived and non‐cancer cultures, using standardized isolation and measurement protocols. To quantitatively describe the data, we defined three interrelated metrics: (i) Protein/particle (PP; pg/particle), representing the protein‐to‐particle ratio of the EV‐enriched isolate; (ii) Vesicle Release Characteristics (VRC; particles/cell/24 h), describing EV release intensity normalized to cell number and time; and (iii) EV Protein Release Characteristics (PRC; pg/cell/24 h), reflecting the amount of protein exported in vesicular form per cell and time unit. Together, this integrated framework allows us to jointly interpret the relationships among EV number, per‐vesicle protein content, and cellular physiological state.

## 2 Materials and Methods

### 2.1 Cell Cultures

#### 2.1.1 Cell Lines and Culturing Conditions

Thirteen cancer and six non‐cancer cell cultures of various tissue origins were used in this study. Cell lines were obtained from our in‐house master cell bank, ECACC or ATCC and were used after thawing at early passages (P3–P6). For each cell culture, six biological replicates were included. The panel was intentionally assembled to include multiple species (human, murine, canine and primate) and diverse tissue origins in order to identify fundamental quantitative features of EV production that are conserved across biological origins, rather than effects restricted to a particular species, lineage or tissue type. Detailed information on cell origin and culture medium composition for each cell line is provided in Table S1.

To ensure optimal viability and minimize pre‐harvest cell death, seeding densities were tailored to each cell culture's growth kinetics, targeting ∼80%–90% confluence at the time of supernatant collection.

All cultures were maintained at 37°C in a humidified incubator with 5% CO_2_ and were supplemented with EV‐depleted fetal bovine serum (FBS; EuroClone, Pero, Italy), and 1% Penicillin–Streptomycin–Amphotericin B mixture (P/S/A; Lonza, Basel, Switzerland), except for the MSC, where MesenCult medium (Stemcell Technologies, Vancouver, British Columbia, Canada) was used without P/S/A.

EV‐depleted FBS was used to minimize contamination from bovine serum–derived vesicles while maintaining physiological culture conditions. This approach was chosen based on our previous findings showing that complete serum deprivation can substantially alter cellular physiology, EV release and proteome composition, thereby substantially influencing EV‐associated protein output (Böröczky 2023).

FBS was prepared by centrifuging at 200,000 × g for 2 h (T‐1270 fixed‐angle rotor in a WX+ ultracentrifuge; both from Sorvall, Thermo Fisher Scientific, Waltham, MA, USA) at 4°C, followed by filtration through a 0.22 µm pore‐size membrane. The depleted FBS was aliquoted and stored at −20°C until use. For detailed culture media and supplements see the Table S1.

#### 2.1.2 Measurements of Cell Number

Cell numbers were determined using a Bürker‐chamber prior to seeding to ensure accurate cell density and after reaching approximately 80%–90% confluence at the time of cell harvest. Cell counting was performed to monitor cell growth and to enable normalization of downstream analyses to cell number where appropriate. Cell viability was assessed in parallel by trypan blue exclusion at harvest. Only supernatants from cultures with ≥90% viability were used for downstream analyses. All cell lines were routinely screened for mycoplasma contamination using Lonza's MycoAlert Mycoplasma Detection Assay, and only mycoplasma‐negative cultures were used.

### 2.2 EV Isolation

#### 2.2.1 Differential Ultracentrifugation (dUC)

(sEV‐enriched fractions were isolated using a combination of filtration and dUC, following the recommendations of the MISEV2023 guidelines (Welsh et al. 2024).

More precisely, cell culture supernatants were collected individually when the cell confluence reached 80–90% confluence (typically after 48 h; in some cases, 48–96 h), centrifuged at 780 × g for 5 min to remove cells, and stored at –80°C. Throughout, cells were cultured in medium supplemented with EV‐depleted FBS as described in Section 2.1.1, and the conditioned medium was harvested as a single collection at the end of the incubation period, with no intermediate medium exchange; the recorded incubation time therefore corresponds to the full EV‐accumulation window used for normalization. Thawed supernatant was centrifuged at 4000 × g for 15 min at 4°C and then filtered through a 0.22 µm pore‐size membrane to remove residual debris and larger vesicles. SEV‐enriched isolates were pelleted by ultracentrifugation at 150,000 × g for 60 min at 4°C using a T‐1270 fixed‐angle rotor in a WX+ ultracentrifuge. The resulting pellets were resuspended in 100 µL of particle‐free PBS (Lonza Group Ltd., Basel, Switzerland).

#### 2.2.2 Size Exclusion Chromatography (SEC)

To assess the robustness and reproducibility of the applied research workflow, a subset of cell lines was randomly selected for repeated analysis, including three non‐cancer cell lines (MDCK, MRC‐5 and VERO E6) and three cancer‐derived cell lines (B16F10, THP‐1, and Ramos). For each of the six selected cell lines, three independent replicates were analyzed using the same experimental and computational workflow.

Following thawing on ice, supernatants (SN) of the 18 samples were centrifuged at 4000 × g for 30 min at 4°C using a fixed‐angle rotor to remove cellular debris. The SN was then transferred into a new tube and the final volume was recorded. Prior to vesicle isolation, 3 mL of SN was concentrated to approximately 1.15 mL using Amicon Ultra‐15 10K centrifugal filters (Merck Millipore) at 4000 × g for 20 min at 4°C.

To remove medium/large EVs, the concentrated samples were centrifuged at 10,000 ×g for 10 min at 4°C. Small EVs were subsequently isolated by SEC using qEV1 Gen2 35 nm columns (Izon Science) according to the manufacturer's recommendations.

Briefly, 1 mL of concentrated sample was loaded onto each column and fractions were collected in 700 µL volumes. Fractions 2–3 were collected for each sample. Following SEC isolation, sEV‐containing fractions were further concentrated using Amicon Ultra 10K centrifugal filters (0.5 mL format). Samples were concentrated stepwise at 14,000 × g for approximately 2 min at 4°C until a final volume of ∼100 µL was reached. Vesicles were recovered by reverse centrifugation at 1000 ×g for 2 min at 4°C, and the final sample volume was adjusted to 100 µL. Purified sEV preparations were stored in low‐binding 1.5 mL tubes at −80°C until downstream analysis.

### 2.3 Measurements of the EV‐Enriched Samples

In accordance with MISEV2023 guidelines, which state that both, the source and the preparation of EVs should be described as quantitatively as possible, we performed a detailed characterization on EV‐enriched samples.

#### 2.3.1 Quantification of the Protein Content

Protease inhibitor cocktail (Roche, Mannheim, Germany) was added to the collected cell culture supernatants prior to centrifugation to prevent proteolytic degradation. Following the isolation, total protein concentration of EV‐enriched preparations was quantified using the bicinchoninic acid (BCA) assay. Protein measurements were performed with the Pierce BCA Protein Assay Kit (Thermo Fisher Scientific, Rockford, IL, USA) according to the manufacturer's instructions. Briefly, samples were incubated with the BCA working reagent at 37°C for 30 min, and absorbance was measured spectrophotometrically, using a benchtop microplate reader (Multiskan RC, Thermo Labsystems, Waltham, MA, USA). Protein concentrations were calculated from a bovine serum albumin standard curve prepared across the 0–2000 µg/mL range, with reliable detection of sample protein concentrations between 20 and 2000 µg/mL.

#### 2.3.2 NTA

To determine the size and amount of particles in the individual EV preparations, NTA was performed using a NanoSight NS300 system equipped with a 532 nm laser (Malvern Panalytical Ltd., Malvern, UK) and NTA software version 3.4. Prior to analysis, samples were diluted in particle‐free PBS. All measurements were conducted under standardized settings: syringe pump flow rate set to 30 units, temperature maintained at 24°C, camera level adjusted to 16, video capture duration of 60 s, with three recordings per sample capturing 30–50 particles/frame. The screen gain was set to 1 and the detection threshold to 4.

#### 2.3.3 Flow Cytometry (FC)

MRC‐5 cell line was selected as a representative model for the characterization, as all samples across the study underwent the same isolation workflow.

Protein surface‐marker profiling of EVs was carried out with the bead‐based, multiplex MACSPlex Exosome Kit (Miltenyi Biotec, Germany) following the manufacturer's instructions. Small EV input was adjusted to 20 µg total protein, as recommended, with the protein content quantified in advance using the previously mentioned Pierce BCA Protein Assay Kit. EV isolates were incubated overnight with dye‐labeled capture beads coated with antibodies against 37 different EV surface antigens on an ES‐30 orbital shaker (Grant Instruments, United Kingdom) at 150 rpm at room temperature (RT). After washing, an APC‐conjugated detection cocktail recognizing tetraspanin CD9, CD63 and CD81 was added for 60 min at RT. Samples were analyzed on a CytoFLEX S flow cytometer (Beckman Coulter, IN, USA); data were acquired with CytExpert v2.4.0.28 and median fluorescence intensities were normalized to the combined APC signal of CD9/CD63/CD81. Representative MACSPlex FC plots are provided in the supplementary materials (Supplementary Document 1), including the capture bead gate, PBS buffer control, MSC EV sample and MSC culture medium control.

To assess the membrane‐enclosed vesicles rather than detergent‐resistant protein complexes, we employed a Calcein AM/Triton X‐100 integrity assay. EV samples were incubated with Calcein AM (1:200 dilution) for 60 min at 37°C, followed by buffer exchange through Amicon 100 kDa filters to remove unbound dye and adjust the volume to 200 µL in particle‐free PBS. Immediately before acquisition, parallel aliquots were treated with 1% Triton X‐100 Surfactant (9410‐OP, Merck Millipore) for 30 min at RT. Detergent treatment lyses lipid bilayers, causing Calcein‐positive EV signals to disappear, whereas Calcein‐negative, detergent‐resistant particles (e.g., protein aggregates) remain detectable. Consistent with current recommendations regarding EV characterization, Triton sensitivity was used as supportive evidence for detergent‐sensitive, membrane‐associated particles, but not as definitive proof of EV specificity. To minimize swarm‐related coincidence detection artifacts and background signal contributions, samples were acquired under diluted conditions, and buffer‐only, unstained, Calcein‐only, and detergent‐treated controls were included throughout the analysis, consistent with current MIFlowCyt‐EV recommendations (Welsh et al. 2020).

#### 2.3.4 Transmission Electron Microscopy (TEM)

Samples were analyzed with a JEM‐1400 Flash transmission electron microscope (JEOL, Tokyo, Japan) to identify their morphological characteristics. To obtain the negatively stained samples, 10 µL of the extract was mounted on a Formvar‐coated 150‐mesh copper grid (Electron Microscopy Sciences, Hatfield, USA). After 1 min, the excessive fluid was blotted away with the edge of a filter paper. The samples were then contrasted with 10 µL 2% uranyl acetate (Electron Microscopy Sciences) in 50% ethanol twice for 2 min each. After the removal of the excessive staining solution, samples were dried under a Petri dish overnight before the electron microscopic evaluation. Negatively stained samples were systematically screened at 5–20,000× magnification to localize the presence of the samples then recorded at 60,000× magnification with a 16 MP Matataki Flash camera (JEOL).

#### 2.3.5 Electron Tomography

For electron tomography, the same samples were analyzed as described in 2.3.4., using a high‐tilt TEM holder head (JEOL). Single TEM images were recorded from ‐44° to +50° tilting angles with a 1° tilting step with Recorder for TEM (System in Frontier, Tokyo, Japan) at 40,000× magnification with the camera described in 2.3.4. Image batch files were saved to tmg file format for reconstruction. Composer (System in Frontier) was used for image alignment; areas with high contrast differences were marked; then cross‐correlation alignment was performed. The reconstructed image block was exported as a binary file and was segmented and colored manually in Visualizer‐Kai (System in Frontier).

**FIGURE 1:**
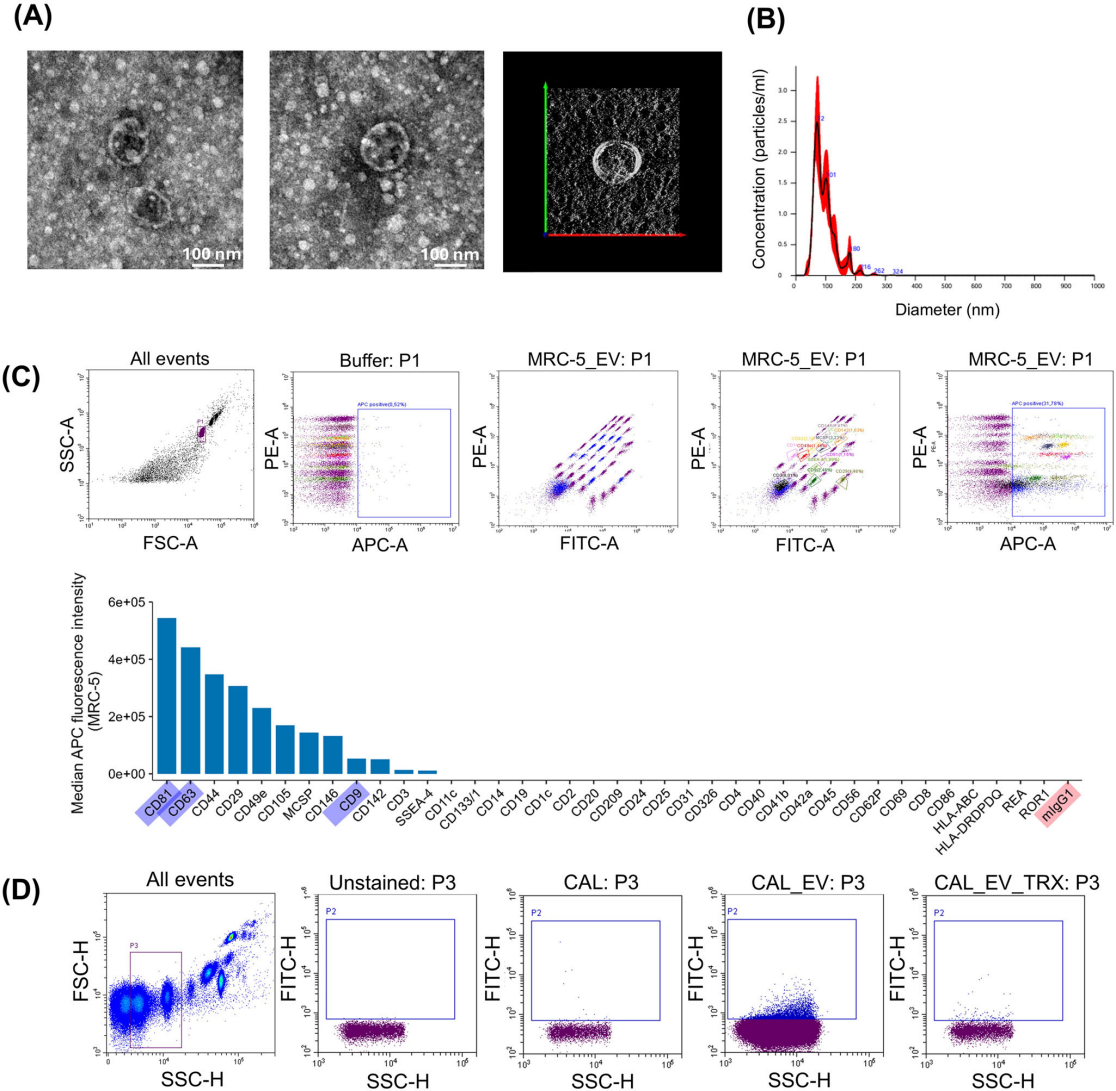
Comprehensive characterization of dUC‐isolated small EVs derived from MRC‐5 cell culture supernatant. (A) TEM and electron tomography images and (B) NTA of MRC‐5 vesicles. (C) Surface‐marker profiling of EV preparations was performed using the MACSPlex Exosome Kit (37 surface antigens). Representative FC plots illustrate the sequential gating strategy and bead‐based detection workflow. The left panel shows the initial FSC/SSC gate used to identify bead populations, followed by the buffer‐only negative control. The subsequent panels show representative fluorescence distributions of antibody‐labeled EV‐bead complexes from MRC‐5‐derived EV preparations across different fluorescence channels. The lower bar graph summarizes the median APC fluorescence intensity (MFI) of the analyzed surface markers. Canonical EV‐associated tetraspanins (CD9, CD63 and CD81) are highlighted in blue, while the isotype control (mIgG1) is shown in red. These measurements were used to confirm the presence of EV‐associated surface markers within the isolated vesicle‐enriched preparations. (D) Calcein‐AM membrane‐integrity assay demonstrates that flow‐cytometric EV events are detergent‐sensitive: Calcein‐positive signals (blue) vanish after treatment with 1% Triton X‐100, confirming their origin from membrane‐enclosed vesicles rather than detergent‐resistant protein complexes. The P3 gate was defined using ApogeeMix sizing beads to capture particles below 300 nm while excluding background noise. The P2 gate was defined as the fluorescence‐positive gate and was set by positioning thresholds above the signals obtained from dye‐only controls. This gate was used to identify Calcein‐positive events. Triton sensitivity was used as supportive evidence for membrane‐enclosed particles, but not as definitive proof of EV specificity.

A summary of the characterization results is provided in Figure 1. In addition, Figure S1 presents representative TEM images of dUC‐isolated EV preparations from additional cell lines, as well as an SEC‐isolated EV preparation showing fewer lipoprotein contaminants.

### 2.4 Statistical Analysis

#### 2.4.1 Variable Construction

The protein amount per particle was characterized using the Protein/particle (PP; pg/particle) variable. This value was calculated from the BCA‐detectable protein concentration measured in the EV‐enriched isolate and the total number of NTA‐detectable particles recovered from the corresponding starting medium volume, according to the following formula:

(1) $$\mathrm{Protein}/\mathrm{particle}(\mathrm{PP})=\frac{\mathrm{Total}\mathrm{protein}[\mathrm{pg}]}{N_{\mathrm{total}\mathrm{particle}}}\;\left[\frac{\mathrm{pg}}{\mathrm{particle}}\right]$$

In the numerator, Total protein [pg] refers to the estimated total amount of protein recovered in the EV‐enriched fraction corresponding to the original medium volume. This value was calculated by multiplying the protein concentration measured in the EV‐enriched isolate by the corresponding medium volume used for EV collection:

(1.1) $$\mathrm{Total}\mathrm{protein}=c_{\mathrm{protein}\left[\frac{\mathrm{pg}}{\mathrm{ml}}\right]}*V_{\mathrm{medium}[\mathrm{ml}]}\;\left[\mathrm{pg}\right]$$

In the denominator, *N*_total particle_– the total number of particles present in the entire medium volume—can be calculated as follows:

(1.2) $$N_{\mathrm{total}\mathrm{particle}}=c_{\mathrm{particle}\left[\frac{\mathrm{particle}}{\mathrm{ml}}\right]}*V_{\mathrm{medium}[\mathrm{ml}]}$$

where *c*_particle_ denotes the particle number concentration (i.e., the number of particles per unit volume) measured by NTA, and *V*_medium_ represents the volume of the culture medium.

To characterize EV release from individual cell cultures, we introduced the Vesicle Release Characteristics (VRC) variable (particles/cell/24 h), which provides an operational estimate of EV release based on the number of particles detected in EV‐enriched preparations, normalized to producer cell number and conditioning time.

(2) $$\begin{array}{ccc} & & \mathrm{Vesicular}\;\mathrm{Release}\;\mathrm{Characteristics}\;(\mathrm{VRC})=\\ & & \frac{N_{\mathrm{total}\;\mathrm{particle}}}{N_{\mathrm{avg}\;\mathrm{cell}}*\left(\mathrm{incubation}\;\mathrm{time}[\mathrm{hours}]/24\right)}\;\left[\frac{\frac{\mathrm{particle}}{\mathrm{cell}}}{24h}\right] \end{array}$$

where *N*_total particle_ denotes the total number of particles present in the entire medium volume, as derived from Equation 1.2.

The denominator includes *N*_avg cell_, which denotes the average cell number over the incubation period and was determined using the following expression:

(2.1) $$N_{\mathrm{avg}\mathrm{cell}}=\frac{N_{\mathrm{final}}-N_{\mathrm{initial}}}{\ln\left(N_{\mathrm{final}}\right)-\ln\left(N_{\mathrm{initial}}\right)}$$

where *N*_initial_ and *N*_final_ represent the cell numbers at the beginning and at the end of the incubation period, respectively. The logarithmically corrected *N*_avg cell_ reduces bias arising from cell growth dynamics (sigmoidal curve), thereby enabling more reliable comparisons of EV production characteristics across different cell lines, even in the presence of differing cell division rates and incubation times.

To characterize the protein output associated with EV release from individual cell cultures, we introduced the Protein Release Characteristics (PRC; pg protein/cell/24 h) metric. PRC provides an operational estimate of this output based on the total detectable protein recovered in EV‐enriched isolates, normalized to producer cell number and conditioning time, and was calculated as follows:

(3) $$\begin{array}{ccc} & & \mathrm{Protein}\;\mathrm{Release}\;\mathrm{Characteristics}\;\left(\mathrm{PRC}\right)=\\ & & \frac{\mathrm{Total}\;\mathrm{protein}\;\left[\mathrm{pg}\right]}{N_{avg\;cell}*\left(\mathrm{incubation}\;\mathrm{time}\;\left[\mathrm{hours}\right]\;/\;24\right)}\;\left[\frac{\frac{\mathrm{pg}}{\mathrm{cell}}}{24\;h}\right] \end{array}$$

where Total protein (pg) is calculated according to Equation 1.1, and *N*_avg cell_ is derived based on Equation 2.1.

The Proliferation rate of the cells was determined as follows:

(4) $$\mathrm{Proliferation}\;\mathrm{rate}\;=\frac{\;\ln\;\left(N_{\mathrm{final}}/N_{\mathrm{initial}}\right)\;}{t}\;\left[\;h^{-1}\right]$$

where N_initial_ and N_final_ denote the cell numbers at the beginning and at the end of the incubation period, respectively, and t represents the incubation time. For example, if a cell culture has a Proliferation rate of 0.015 h^−^^1^, this corresponds to a 1.5% increase in cell number per hour.

Finally, to characterize the relationship between particle number and protein content in EV‐enriched isolates using a single metric, we calculated the number of NTA‐detectable particles per unit of BCA‐detectable protein as follows:

(5) $$\mathrm{Particle}/1\mathrm{pg}\mathrm{protein}=\frac{\mathrm{VRC}\;\left[\mathrm{particle}/\mathrm{cell}/24h\right]}{\mathrm{PRC}\;\left[\mathrm{pg}/\mathrm{cell}/24h\right]}\;\mathrm{or}\frac{1}{\mathrm{PP}}$$

This ratio indicates how many particles correspond to a given amount of protein in an EV‐enriched isolate. It therefore describes the relationship between measured particle abundance and total recovered protein.

#### 2.4.2 Comparison of Proliferation Rate and EV‐Associated Metrics Between Cancer and Non‐Cancer Groups

Statistical comparisons of proliferation‐ and EV‐related parameters between non‐cancer and cancer‐derived cell lines, as well as the exploration of relationships among proliferation, vesicle size, measured protein content of EV‐enriched isolates, were performed using cell line–level analyses.

Proliferation rates and EV‐associated metrics of the non‐cancer and cancer‐derived groups were compared using generalized linear models (GLMs) to appropriately handle distributional skewness. Given the approximately symmetric distribution of the Proliferation rate, this variable was analyzed using a linear model assuming a normal distribution (Gaussian family, identity link). In contrast, EV‐related quantitative variables (PP, VRC, PRC) exhibited right‐skewed distributions and took exclusively positive values; therefore, GLMs assuming a gamma distribution with a log link function were fitted for these outcomes. Estimated marginal means and their 95% confidence intervals were used for visualization.

These analyses were performed for all dUC‐isolated samples and for the subset of six cell lines randomly selected for SEC isolation. Given the reduced sample size of the SEC dataset, trends in SEC isolates were interpreted primarily based on fold‐change estimates and their confidence intervals. Thus, comparisons between dUC and SEC isolates focused on the direction, magnitude and uncertainty of the estimated effects rather than on formal significance testing alone.

#### 2.4.3 Investigation of the Role of Vesicle Size in Shaping EV Protein Content

To assess whether differences between cancer‐derived and non‐cancer cell lines could be explained by differences in particle size, vesicle diameter (particle diameter, nm) was included in the analysis. Vesicle size was characterized at the cell line level using the mode of the particle size distribution obtained by NTA.

As a first step, differences in vesicle diameter between the two groups were tested using a GLM with a gamma distribution and log link. Subsequently, the relationship between particle size and protein‐based EV parameters (PP, PRC) was examined at the cell line level using partial Pearson correlation, with cancer/non‐cancer status treated as a control variable. This approach allowed the evaluation of the size–protein content relationship independently of cancer origin. Confidence intervals for the partial correlation coefficients were estimated using a bootstrap procedure with 5000 resamples.

#### 2.4.4 Analysis of the Relationship Between Proliferation and EV Parameters

Prior to the analyses presented in Sections 2.4.3–2.4.6, variable distributions were assessed, and logarithmic transformation was applied in cases of skewed distributions. Subsequently, z‐score standardization was performed to place variables on a comparable scale. This standardization was also justified from an interpretative perspective, as a value of ‘0’ on the original measurement scale does not represent a biologically meaningful baseline in several cases (e.g., a Proliferation rate of 0 is not interpretable for viable cells), which would have complicated the interpretation of regression results. Transformed variables were denoted using the prefix ‘Scaled’ (e.g., Scaled Proliferation rate).

The relationship between proliferation and EV parameters PP, PRC) was analyzed using Pearson correlation, comparing the Scaled Proliferation rate with Scaled PP and Scaled PRC values, respectively.

This analysis was performed on all samples isolated using dUC, as well as on the six cell lines that were randomly selected for SEC isolation.

#### 2.4.5 Assessment of the Independent Effects of Cancer Origin and Proliferation on Protein‐to‐Particle Ratio of EV‐Enriched Isolates

To identify the determinants of the Scaled PP variable, linear regression models were fitted using a hierarchical (stepwise) approach. Specifically, ordinary least‐squares models were fitted with Scaled PP as the dependent variable. **Model 1** included only the Group (cancer origin) predictor, whereas **Model 2** additionally incorporated the Scaled Proliferation rate variable.

This structure allowed evaluation of the extent to which cancer origin and proliferation explain variance in Scaled PP, and whether their effects can be considered independent. Group was coded, with non‐cancer‐derived cell lines used as the reference category and cancer‐derived cell lines as the comparison category.

In the regression tables, Coeff denotes the estimated regression coefficients, SE their standard errors, and Lower/Upper the bounds of the 95% confidence intervals. Model explanatory power was characterized using the coefficient of determination (*R* ^2^); comparison of *R* ^2^ values across models quantified the increase in explained variance attributable to the inclusion of proliferation.

The results of the model‐based estimates could not be compared between the SEC and dUC isolates, because the complexity of the model exceeded the available sample size.

#### 2.4.6 Investigation of the Relationship Between Estimated EV Release and Protein Recovery in EV‐Enriched Isolates Differs by Cancer Origin

To examine the relationship between protein recovered in EV‐enriched isolates per cell (Scaled PRC; pg protein/cell/24 h) and estimated EV release (Scaled VRC), a linear regression model was fitted with Scaled PRC as the dependent variable and Group, Scaled VRC, and their interaction (Group × Scaled VRC) as predictors.

The interaction term tested whether the direction and/or strength of the VRC–PRC relationship differed between non‐cancer and cancer‐derived cell lines. The model was fitted assuming a Gaussian family with an identity link.

The categorical Group variable was coded using treatment contrasts, with the non‐cancer group as the reference category; accordingly, the interaction coefficient reflected how the relationship between scaled VRC and PRC differed between cancer‐derived and non‐cancer cell lines. Model explanatory power was reported using *R* ^2^.

#### 2.4.7 Comparison of Particle Numbers Per Unit of Protein in EV‐Enriched Isolates

To assess whether EV‐enriched isolates from cancer‐derived and non‐cancer cell lines contain different numbers of particles per unit of protein, the particle/pg protein metric was used. Group comparisons were performed using GLM (gaussian family, identity link).

To determine whether the observed difference was independent of the species composition of the cell‐line panel, an exact within‐species stratified permutation test was additionally performed on log10‐transformed particle/pg protein values. In this analysis, the algorithm reassigned the cancer and non‐cancer labels among cell lines belonging to the same species, while preserving the original number of samples in each group. Thus, human cell lines were reassigned only among human cell lines and murine cell lines only among murine cell lines, ensuring that the species composition remained unchanged in every alternative grouping.

For each possible reassignment, the cancer–non‐cancer difference was recalculated. If the observed difference were primarily caused by species origin, differences of a similar magnitude would occur frequently after label reassignment, resulting in a relatively high, non‐significant *p*‐value. Conversely, if similarly large differences occurred only rarely, the resulting low *p*‐value would indicate that the finding could not be explained by species composition alone. Human and murine cell lines were the informative strata, and all 990 possible species‐preserving assignments were evaluated using a two‐sided exact test.

All *p*‐values were calculated based on two‐sided tests, and the significance level was set to α = 0.05 in all analyses. Statistical analyses and figure generation were performed using JASP version 0.95.3. (JASP Team 2025)

## 3 Results

### 3.1 Estimated EV Release and Particle‐to‐Protein Ratios of EV‐Enriched Isolates Differ Markedly Between Non‐Cancer and Cancer Cell Lines

The first step of the analysis aimed to provide an overall overview: we examined how the proliferation and vesicular parameters of the investigated cell lines differ between the cancer and non‐cancer groups, and how the distributions of these parameters are shaped within the two groups.

**FIGURE 2:**
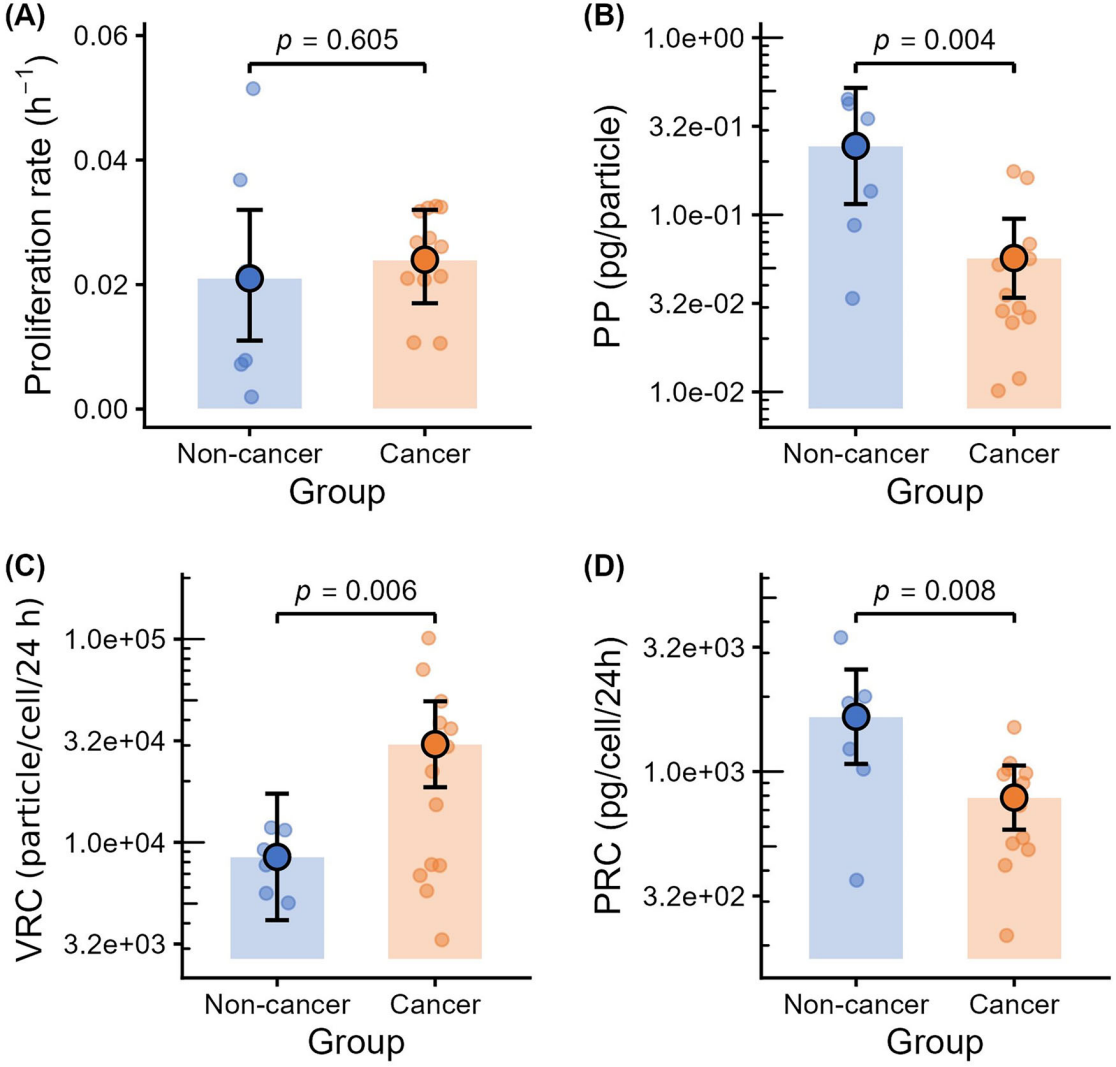
Comparison of proliferation rate and particle‐related parameters in non‐cancer and cancer cell lines. Bars show the generalized linear model‐derived estimated marginal means for (A) Proliferation rate, (B) average protein content per particle (PP, pg/particle), (C) Vesicle Release Characteristics (VRC, particles/cell/24 h), and (D) Protein Release Characteristics (PRC, pg/cell/24 h) in non‐cancer (blue) and cancer (orange) cell lines. Error bars indicate the corresponding 95% confidence intervals. Individual points represent single cell lines. Model‐based *p*‐values are shown above each panel.

Proliferation rate, average protein content per particle (PP, pg/particle), estimated EV release based on the number of particles recovered per cell over 24 h (VRC, particles/cell/24 h), and the amount of protein recovered in EV‐enriched isolates per cell over 24 h (PRC, pg protein/cell/24 h) were determined for each cell line. Differences between groups were assessed using GLMs (Figure 2).

Based on the Proliferation rate, no clear separation is observed between cancer and non‐cancer cell lines (*p* = 0.605) (Figure 2A). The estimated mean Proliferation rate of non‐cancer cell lines was 0.021 h^−^^1^ (95% CI: 0.011–0.032 h^−^^1^), whereas cancer cell lines exhibited an estimated marginal mean of 0.024 h^−^^1^ (95% CI: 0.017–0.032 h^−^^1^), indicating a somewhat higher value in the cancer group. However, the confidence intervals overlap substantially.

In contrast, PP shows a pronounced difference between the two groups (Figure 2B). In non‐cancer cell lines, the mean PP value was 0.245 pg/particle (95% CI: 0.115–0.521 pg/particle), whereas in cancer cell lines it was 0.057 pg/particle (95% CI: 0.034–0.095 pg/particle). Accordingly, EV‐enriched isolates from cancer cells generally exhibit a lower protein‐to‐particle ratio (p = 0.004).

With respect to VRC, cancer status was associated with a difference in the opposite direction (Figure 2C). The estimated mean VRC of non‐cancer cell lines was 8483 particles/cell/24 h (95% CI: 4145–17,362), whereas cancer cell lines exhibited an estimated mean of 30,400 particles/cell/24 h (95% CI: 18,688–49,452; *p* = 0.006).

Consistent with these findings, PRC shows lower values in cancer cell lines (Figure 2D). The mean PRC of non‐cancer cell lines was 1662 pg/cell/24 h (95% CI: 1073–2573 pg/cell/24 h), whereas cancer cell lines exhibited 786 pg/cell/24 h (95% CI: 584–1058 pg/cell/24 h). Accordingly, despite showing higher estimated EV release, cancer cell lines had lower amounts of protein recovered in EV‐enriched isolates per cell over a 24‐h period (*p* = 0.008).

Taken together, these results reveal a distinct quantitative pattern in EV‐enriched isolates from cancer and non‐cancer cell lines. Cancer cell lines showed 3.6‐fold higher estimated EV release, while their EV‐enriched isolates exhibited a 4.3‐fold lower protein‐to‐particle ratio. At the same time, the amount of protein recovered in EV‐enriched isolates per cell was 2.1‐fold lower in cancer cell lines. Thus, cancer‐derived isolates were characterized by more detected particles but less recovered protein, both per particle and per producer cell. These findings describe a reproducible difference in the particle–protein relationship of EV‐enriched isolates, but do not establish that individual cancer‐derived EVs contain less protein.

To determine whether the dUC‐based observations were also reproduced using a method expected to yield higher‐purity isolates, the same six cell lines were processed in parallel by dUC and SEC. We then compared the particle‐related parameters between the two sets of EV‐enriched isolates. As this subset included three non‐cancer and three cancer‐derived cell lines, group differences did not reach statistical significance within either isolation method when analyzed separately. Therefore, rather than relying solely on significance testing, we focused on model‐estimated group differences, expressed as Cancer / Non‐cancer fold changes with 95% confidence intervals, to compare the direction, magnitude, and uncertainty of the observed trends across isolation methods (see Figure S2).

For PP, cancer‐derived cell lines showed lower values in EV‐enriched isolates obtained using both isolation methods. The Cancer / Non‐cancer fold‐change was 0.19 for EV‐enriched isolates obtained by SEC (95% CI: 0.02–1.62) and 0.11 for EV‐enriched isolates obtained by dUC (95% CI: 0.03–0.44), showing concordant effect directions across the two isolation approaches.

For VRC, the opposite pattern was observed. Cancer‐derived cell lines showed higher estimated EV release. The Cancer / Non‐cancer fold‐change in VRC was 5.71 for EV‐enriched isolates obtained by SEC (95% CI: 1.05–30.97) and 7.12 for EV‐enriched isolates obtained by dUC (95% CI: 2.57–19.68), again indicating a consistent direction of effect across methods.

For PRC, cancer‐derived cell lines showed slightly lower values with both isolation methods. The Cancer / Non‐cancer fold‐change was 0.79 for EV‐enriched isolates obtained by SEC (95% CI: 0.29–2.14) and 0.70 for EV‐enriched isolates obtained by dUC (95% CI: 0.41–1.19), indicating that the higher estimated EV release observed in cancer‐derived cell lines was not accompanied by a higher amount of protein recovered in EV‐enriched isolates per cell.

**Taken together, the SEC‐based results supported the dUC‐based observations**. Across both isolation methods, cancer‐derived cell lines consistently showed higher estimated EV release based on particle recovery, lower protein‐to‐particle ratios in EV‐enriched isolates, and slightly lower amounts of protein recovered in EV‐enriched isolates per cell.

### 3.2 Differences in Protein‐to‐Particle Ratios Between Cancer‐ and Non‐Cancer‐Derived Isolates Are Not Explained by Particle Size

Based on the results presented above, EV‐enriched isolates from cancer cell lines showed lower PP (pg/particle), whereas cancer cell lines showed higher VRC (particles/cell/24 h). A possible explanation for this pattern could be that differences in the size of the detected particles contribute to the lower PP observed in cancer‐derived isolates.

To assess this possibility, we first examined whether the modal diameter of NTA‐detectable particles differed between cancer and non‐cancer cell lines.

**FIGURE 3:**
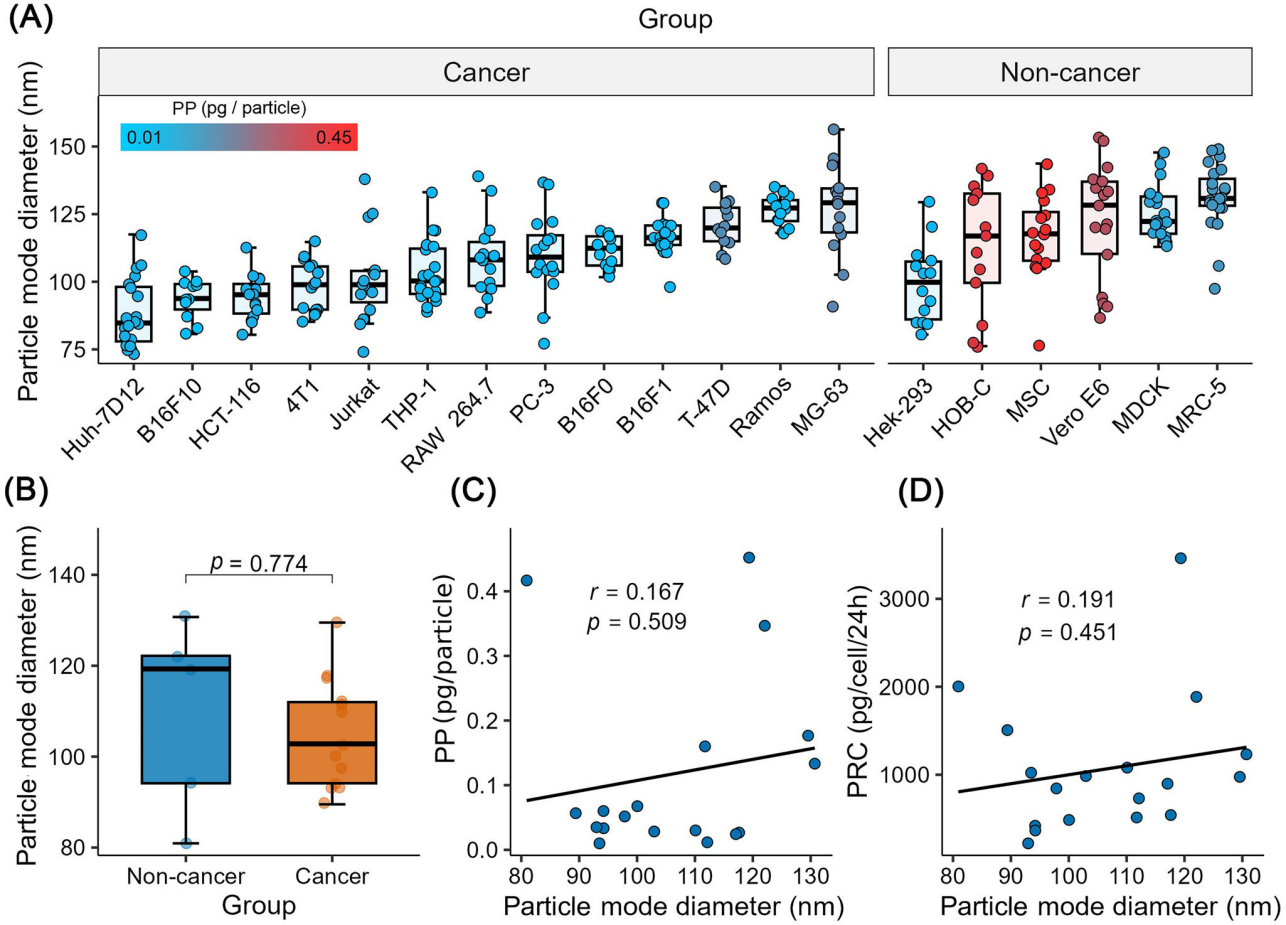
Comparison of particle size with PP and PRC. (A) Boxplots shows the Particle mode diameter (nm) distribution for all cell lines (before averaging the nanoparticle‐tracking analysis results). Blue‐to‐red gradient indicates the Protein/particle (PP, pg/particle) values. (B) Boxplot shows the Particle mode diameter (nm) distribution in the non‐cancer and cancer group. Group differences were assessed using GLMs. (C) Scatter plots show the relationship between Particle mode diameter (nm) and PP (pg/particle), or (D) Particle mode diameter and Protein Release Characteristics (PRC, pg/cell/24 h). Each point represents a single cell line. *P*‐value originates from partial correlation analysis (the trend line is only for illustration purposes, not for inference, therefore no error bar is visible).

Although individual cell lines showed heterogeneity (Figure 3A), no significant difference in particle size was observed at the group level between cancer and non‐cancer cell lines (*p* = 0.774) (Figure 3B).

We subsequently investigated whether, at the level of individual cell lines, particle size was associated with PP or PRC values.

Particle size showed no significant correlation with either PP (*r* = 0.167, 95% CI: −0.326 to 0.588; *p* = 0.509) (Figure 3C) or PRC (*r* = 0.191, 95% CI: −0.304 to 0.603; *p* = 0.451) (Figure 3D).

These results provide no evidence that the differences in PP observed between EV‐enriched isolates from cancer and non‐cancer cell lines are attributable to differences in particle size.

### 3.3 Proliferation Rate Is Inversely Associated With Protein‐to‐Particle Ratios and Per‐Cell Protein Recovery in EV‐Enriched Isolates

In the next step, the focus shifted from direct group comparisons to examining the relationship between cellular proliferation rate and both the protein‐to‐particle ratio (PP, pg/particle) of EV‐enriched isolates and the amount of protein recovered in EV‐enriched isolates per cell over 24 h (PRC; pg protein/cell/24 h).

**FIGURE 4:**
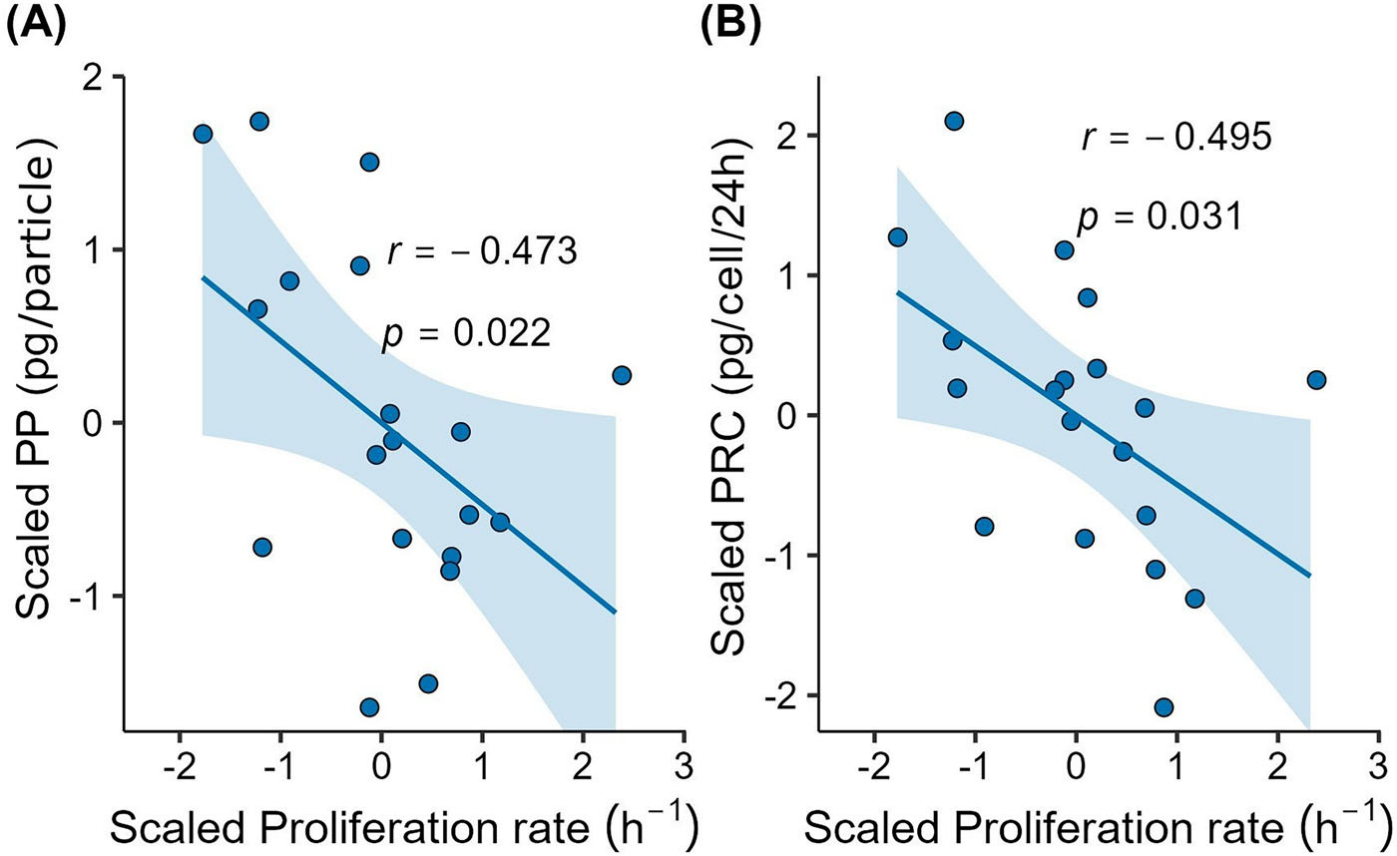
Correlations between proliferation and protein‐related parameters. Scatter plots with linear regression lines and 95% confidence bands illustrate the relationships between log‐transformed, z‐standardized variables. (A) Scaled PP (pg/particle) as a function of scaled Proliferation rate (h^−^^1^). (B) Scaled PRC (pg/cell/24 h) as a function of scaled Proliferation rate (h^−^^1^). Each point represents a single cell line. Pearson's correlation coefficient (r) and the corresponding p‐value derived from the correlation test are shown within both panels.

A significant negative correlation was observed between Proliferation rate and PP (*r* = −0.473; 95% CI: −0.763 to −0.124; *p* = 0.022), indicating that EV‐enriched isolates from more rapidly proliferating cell lines tended to exhibit lower PP values (Figure 4A). (Figure 4A). Consequently, higher proliferation rates were associated with lower PRC values, reflecting lower per‐cell protein recovery in EV‐enriched isolates (*r* = −0.495 [95% CI: −0.775–0.153], *p* = 0.031) (Figure 4B).

Overall, the correlation analyses revealed a coherent pattern in which proliferation rate was associated with the particle–protein characteristics of EV‐enriched isolates: rapidly dividing cell lines showed lower PP ratios and lower PRC values, whereas more slowly proliferating cell lines tended to show higher values for both parameters.

To assess whether the proliferation‐associated PP and PRC patterns were comparable between isolation methods, we analyzed the six cell lines processed in parallel by SEC and dUC (Figure S3).

The relative positioning of the individual cell lines was similar between the two methods for both PP and PRC, indicating comparable cell‐line‐specific particle‐protein. However, when the analysis was restricted to this paired six‐cell‐line subset, no significant correlation was detected between Proliferation rate and either PP or PRC with either isolation method. For PP, the correlation was not significant in SEC (*r* = 0.430, *p* = 0.395) or dUC (*r* = −0.086, *p* = 0.871). Similarly, PRC showed no significant association with Proliferation rate in SEC (*r* = 0.263, *p* = 0.615) or dUC (*r* = −0.152, *p* = 0.774).

Thus, the SEC–dUC comparison supports the comparability of the two isolation approaches at the level of relative cell‐line profiles, while the proliferation‐associated correlations observed in the full dUC dataset were not detectable in the restricted six‐cell‐line subset.

### 3.4 Cancer Origin and Proliferation Are Independently Associated With Lower Protein‐to‐Particle Ratios in EV‐Enriched Isolates

Based on the preceding results, the average protein‐to‐particle ratio (PP, pg/particle) is associated with both cancer origin and proliferation. Accordingly, we applied a series of nested models to assess the extent to which of these factors act jointly and the degree to which they independently influence protein to particle ratio, as specified in Section 2.4.5. The estimated model coefficients are summarized in Table 1.

**TABLE 1:** Linear models examining how the PP values relate to cancer group, Proliferation rate and estimated EV release. Model 1 includes only cancer versus non‐cancer status; Model 2 adds the scaled Proliferation rate; Coefficients (Coeff) indicate the direction and strength of association with PP (negative values indicate lower protein‐to‐particle ratios with higher predictor values or in cancer‐derived lines). SE denotes standard error, *p* the significance level, and Lower/Upper the bounds of the 95% confidence interval.

Model	Variable	Coeff	SE	*p*	95% CI	
					Lower	Upper
1	Group = Cancer	−1.283	0.401	0.005	−2.13	−0.436
2	Group = Cancer	−0.969	0.387	0.024	−1.789	−0.148
	Scaled Proliferation rate (h^−1^)	−0.415	0.185	0.039	−0.807	−0.023

In the first step, only cancer versus non‐cancer origin was included in the model (Table 1, **Model 1)**. In this comparison, cancer cell lines were consistently associated with lower PP values, indicating that EV‐enriched isolates derived from cancer cells are markedly more protein‐poor than those from non‐cancer cell lines (*p* = 0.005). Overall, cancer versus non‐cancer origin accounted for 33.9% of the differences in PP observed between cell lines (*R* ^2^ = 0.339).

In the subsequent step (Table 1, **Model 2**), Proliferation rate was included alongside cancer origin. The results indicated that more rapidly dividing cell lines systematically showed lower PP ratios in their EV‐enriched isolates (*p* = 0.039). Although the effect associated with cancer origin was attenuated, it remained statistically significant (*p* = 0.024). This suggests that the lower PP ratio observed in cancer‐derived cell lines are partly associated with accelerated proliferation, while cancer origin remains independently associated with lower PP ratio. When both proliferation and cancer origin were taken into account, the model accounted for more than half of the between‐cell line variation in PP (*R* ^2^ = 0.525; 52.5%).

Together, these findings indicate that the lower PP values, reflecting lower protein‐to‐particle ratios in EV‐enriched isolates, observed in cancer‐derived cell lines are partly associated with faster proliferation, while cancer origin also remains associated with this lower ratio independently of proliferation.

### 3.5 Higher Estimated EV Release Is Associated With Higher PRC in Cancer But Not in Non‐Cancer Cell Lines

We first observed that cancer cell lines showed higher estimated EV release (VRC; particles/cell/24 h) compared with non‐cancer cell lines. At the same time, EV‐enriched isolates from cancer cell lines had lower protein‐to‐particle ratios (PP, pg/particle), and cancer cell lines showed lower per‐cell protein recovery in EV‐enriched isolates (PRC; pg protein/cell/24 h).

These findings raise the question of whether the lower PP ratios observed in cancer‐derived isolates are primarily associated with cancer origin itself or partly reflect a ‘dilution effect’ resulting from increased VRC. Moreover, we sought to determine whether PRC remains relatively stable despite increasing VRC, resulting in lower PP ratios, or whether higher VRC is accompanied by a corresponding increase in PRC.

To address these questions, we examined the relationship between VRC and PRC and assessed whether this relationship differed between cancer and non‐cancer cell lines. Specifically, we fitted a series of models in which PRC was expressed as a function of cancer versus non‐cancer status, VRC, and the interaction between these two factors, as specified in Section 2.4.6 (Table 2, **Models 1–3**).

**TABLE 2:** Linear models examining how the PRC values relate to cancer/non‐cancer origin and VRC. Model M_1_ includes only scaled VRC , M_2_ adds the Group, and M_3_ includes Group and VRC interaction. Coefficients (Coeff) indicate the expected change in standardized PRC associated with each predictor (negative values denote lower protein content per particle in cancer‐derived lines or at higher predictor values). SE denotes standard error, *p* the significance level, and Lower/Upper the bounds of the 95% confidence interval.

Model	Variable	Coeff	SE	*p*	95% CI	
					Lower	Upper
1	Scaled VRC	0.048	0.242	0.847	−0.464	0.559
2	Scaled VRC	0.292	0.231	0.225	−0.198	0.783
	Group = Cancer	−1.235	0.485	0.021	−2.263	−0.208
3	Scaled VRC	−2.149	0.930	0.036	−4.131	−0.166
	Group = Cancer	0.184	0.670	0.787	−1.244	1.611
	Scaled VRC × Group	2.555	0.952	0.017	0.527	4.583

The model explains approximately 52% of the between–cell line variation in PRC (*R* ^2^ = 0.521; *p* = 0.010), indicating that VRC and cell type group membership (cancer versus non‐cancer) are jointly associated with differences in PRC.

In Model M1, VRC alone explained essentially none of the variation in PRC (0.20% explained; *R* ^2^ = 0.002), and VRC was not associated with PRC (*p* = 0.847), indicating that VRC alone does not predict PRC across the full panel of cell lines.

When Group was added (Table 2, Model M2), the explained variance increased to 29.00% (*R* ^2^ = 0.290). In this model, Group was significantly associated with PRC (*p* = 0.021), with cancer cell lines showing lower PRC than non‐cancer cell lines, while VRC remained non‐significant (*p* = 0.225).

**FIGURE 5:**
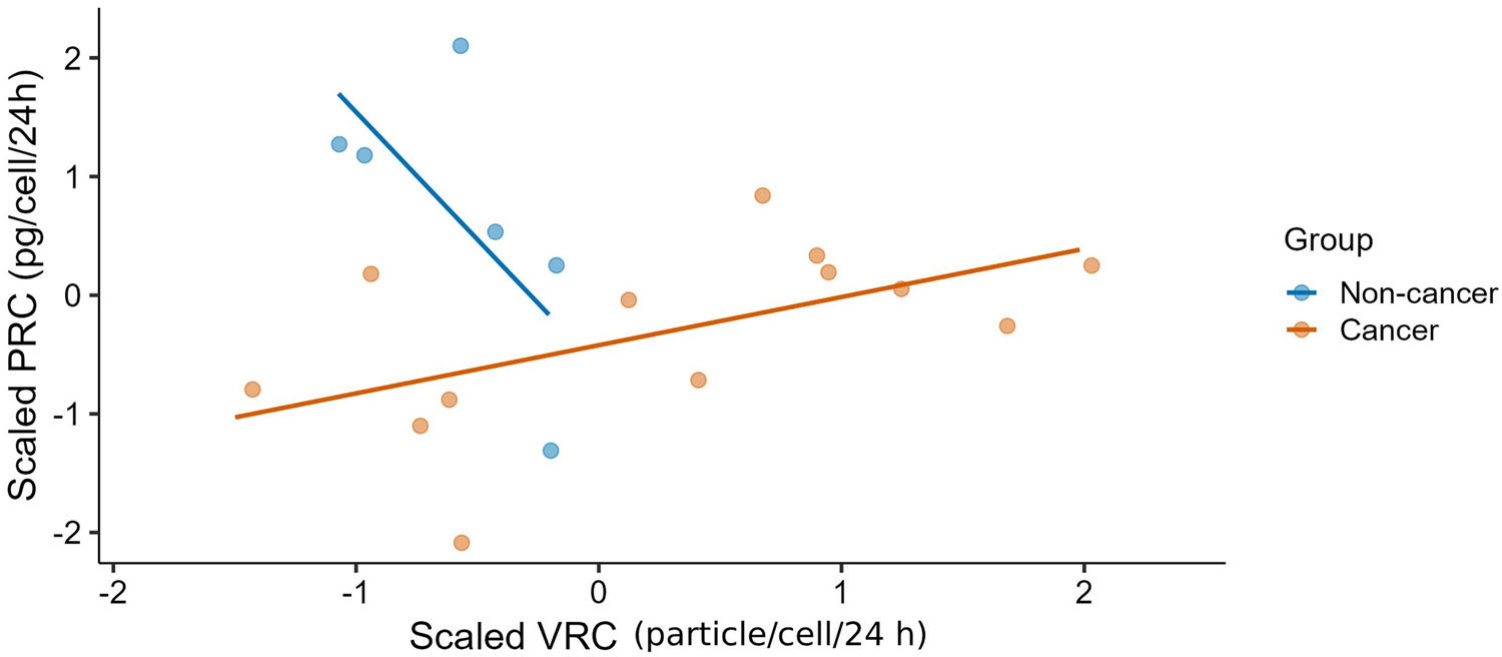
Association of estimated EV release (VRC) and cancer status with per‐cell protein recovery in EV‐enriched isolates (PRC). It illustrates the interaction between scaled VRC and cancer status in relation to scaled PRC. Each point represents a single cell line, separated by group (non‐cancer: blue; cancer: orange). Separate regression lines are shown for the two groups. In non‐cancer cell lines, higher VRC is associated with lower PRC values, whereas in cancer‐derived cell lines higher VRC corresponds to higher PRC values. These divergent slopes visualize the significant Group × VRC interaction identified in the regression model.

Allowing the VRC–PRC relationship to differ by Group (Table 2, **Model M3**) increased the explained variance further to 52.10% (*R* ^2^ = 0.521) and revealed a significant VRC × Group interaction (*p* = 0.017), demonstrating that the relationship between estimated EV release and per‐cell protein recovery in EV‐enriched isolates differs between cancer and non‐cancer cell lines. In the non‐cancer group, higher VRC was associated with lower PRC (*p* = 0.036), indicating that in non‐cancer cells higher estimated EV release is accompanied by lower per‐cell protein recovery in EV‐enriched isolates. In contrast, in cancer cell lines the VRC–PRC relationship was shifted upward relative to non‐cancer (i.e., the negative association was attenuated and tended towards the opposite direction), consistent with the interpretation that in cancer cells higher estimated EV release is not associated with lower PRC but instead with maintained or increased PRC as VRC rises (Figure 5).

In biological terms, these findings suggest that the two cell groups show distinct relationships between estimated EV release and per‐cell protein recovery in EV‐enriched isolates. Non‐cancer cell lines showed lower VRC and higher PP values, while increasing VRC was associated with lower PRC. In contrast, cancer cell lines showed higher VRC and lower PP values, and increasing VRC was associated with higher PRC.

This pattern indicates that in non‐cancer cells higher estimated EV release is not accompanied by maintained or increased PRC, whereas in cancer cells PRC appears to increase in parallel with VRC.

### 3.6 Equal Protein Doses of Cancer‐Derived Isolates Contain More Particles Than Non‐Cancer‐Derived Isolates

The results obtained so far indicate that the particle‐ and protein‐related characteristics of EV‐enriched isolates from cancer and non‐cancer cell lines differ at multiple levels: cancer cell lines show higher estimated EV release (VRC; particles/cell/24 h), while their EV‐enriched isolates exhibit lower protein‐to‐particle ratios (PP; pg protein/particle) and lower per‐cell protein recovery (PRC; pg protein/cell/24 h) than those from non‐cancer cell lines. Because these features are interrelated, it became necessary to examine a metric that captures them jointly.

**FIGURE 6:**
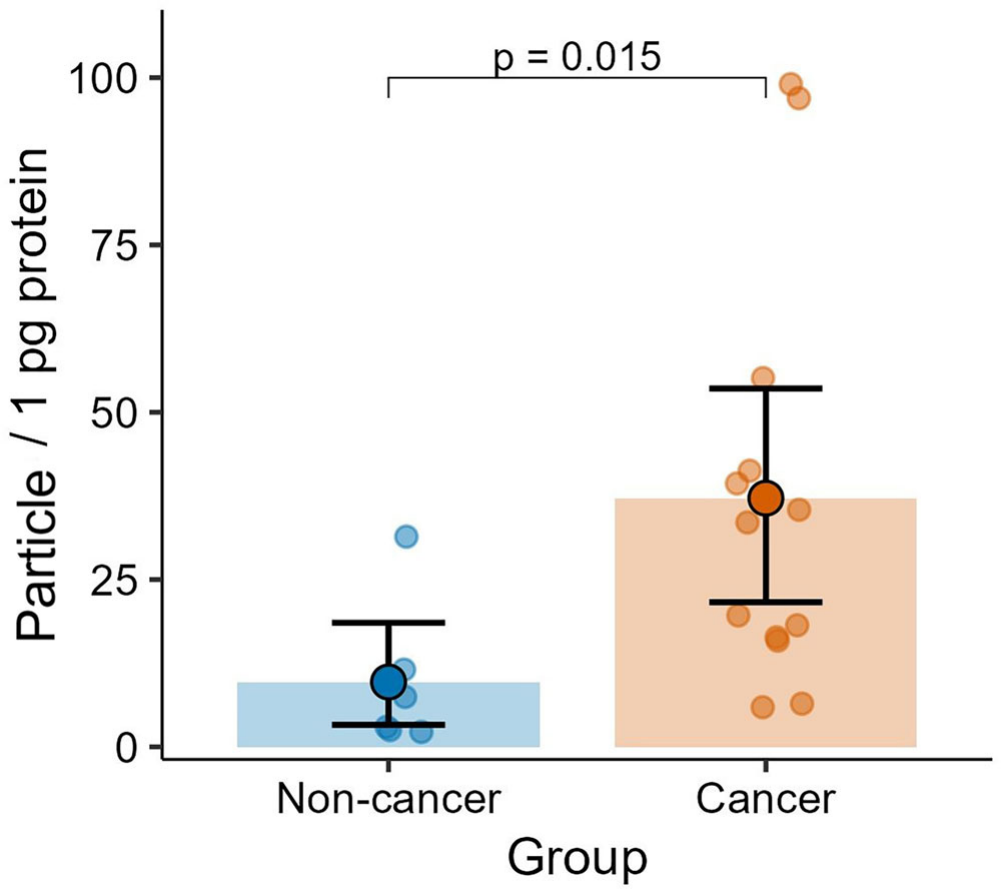
Particle number per unit vesicular protein in non‐cancer and cancer‐derived cell lines. The figure compares the number of particles per picogram of protein in EV‐enriched isolates between non‐cancer cell lines (blue) and cancer‐derived cell lines (orange). Each point represents a single cell line. Bars indicate the mean, and error bars show the corresponding 95% confidence intervals.

The particles/pg protein ratio expresses how many NTA‐detectable particles correspond to a unit amount of BCA‐detectable protein in an EV‐enriched isolate, thereby reflecting the combined behaviour of measured particle abundance and recovered protein content (Figure 6).

Cancer‐derived cell lines exhibited a significantly higher number of particles per picogram of protein in EV‐enriched isolates than non‐cancer cell lines. The mean particle‐to‐protein ratio was 37.160 particles/pg protein in cancer‐derived cell lines (*n* = 13; 95% CI: 18.672–55.649), compared with 9.692 particles/pg protein in non‐cancer cell lines (*n* = 6; 95% CI: −2.109–21.492). The mean difference was 27.469 particles/pg protein in favour of the cancer‐derived cell lines (95% CI: 7.079–47.858; *p* = 0.015).

We assessed whether the observed difference in particles per pg protein was generalizable across the diverse biological origins represented in the panel. To distinguish a broadly conserved pattern from a pattern driven by the particular species composition of the dataset, we performed a species‐preserving permutation test (see Section 2.4.7 for methodological details). Of the 990 alternative group assignments that retained the original species structure, only 19 produced a difference as large as or larger than that observed in the original data (*p* = 0.019). These results support that the observed difference is not driven by a single species origin and is consistently detectable across the biologically diverse cell‐line panel.

This difference is consistent with the preceding results: the combination of higher estimated EV release and lower protein‐to‐particle ratios in cancer cell lines is reflected in a higher particles/pg protein ratio, whereas the opposite pattern observed in non‐cancer cell lines is associated with a lower ratio.

### 3.7 Practical Framework for Particle‐ and Protein‐Based Dose Normalization in EV‐Enriched Isolates

Our results show that particle number and protein content in EV‐enriched isolates do not provide interchangeable information. Because PP differed systematically between cancer and non‐cancer cell lines, matching EV‐enriched isolates by only one of these quantities will necessarily mismatch the other.

Using the estimated mean PP values from Figure 2B, a fixed dose of 10 µg protein corresponds to 4.26 × 10^7^ particles for non‐cancer‐derived isolates and 1.86 × 10^8^ particles for cancer‐derived isolates, that is, a 4.37‐fold difference in particle number. Conversely, a fixed dose of 1 × 10^8^ particles corresponds to 23.5 µg protein for non‐cancer‐derived isolates and 5.37 µg for cancer‐derived isolates, again a 4.37‐fold difference in the opposite direction. Thus, neither protein‐only nor particle‐only normalization is sufficient when PP differs between EV‐enriched isolates.

Based on this, we recommend a simple multidimensional reporting framework. For production studies involving EV‐enriched isolates, we recommend reporting two output metrics: estimated EV release per cell and time (VRC) and protein recovery in EV‐enriched isolates per cell and time (PRC). We further recommend reporting the protein‐to‐particle ratio of EV‐enriched isolates (PP), or equivalently its reciprocal, particles per unit protein, to link these two dimensions. For functional or therapeutic experiments, we recommend reporting both the applied particle number and the applied protein amount together with the corresponding PP value.

**FIGURE 7:**
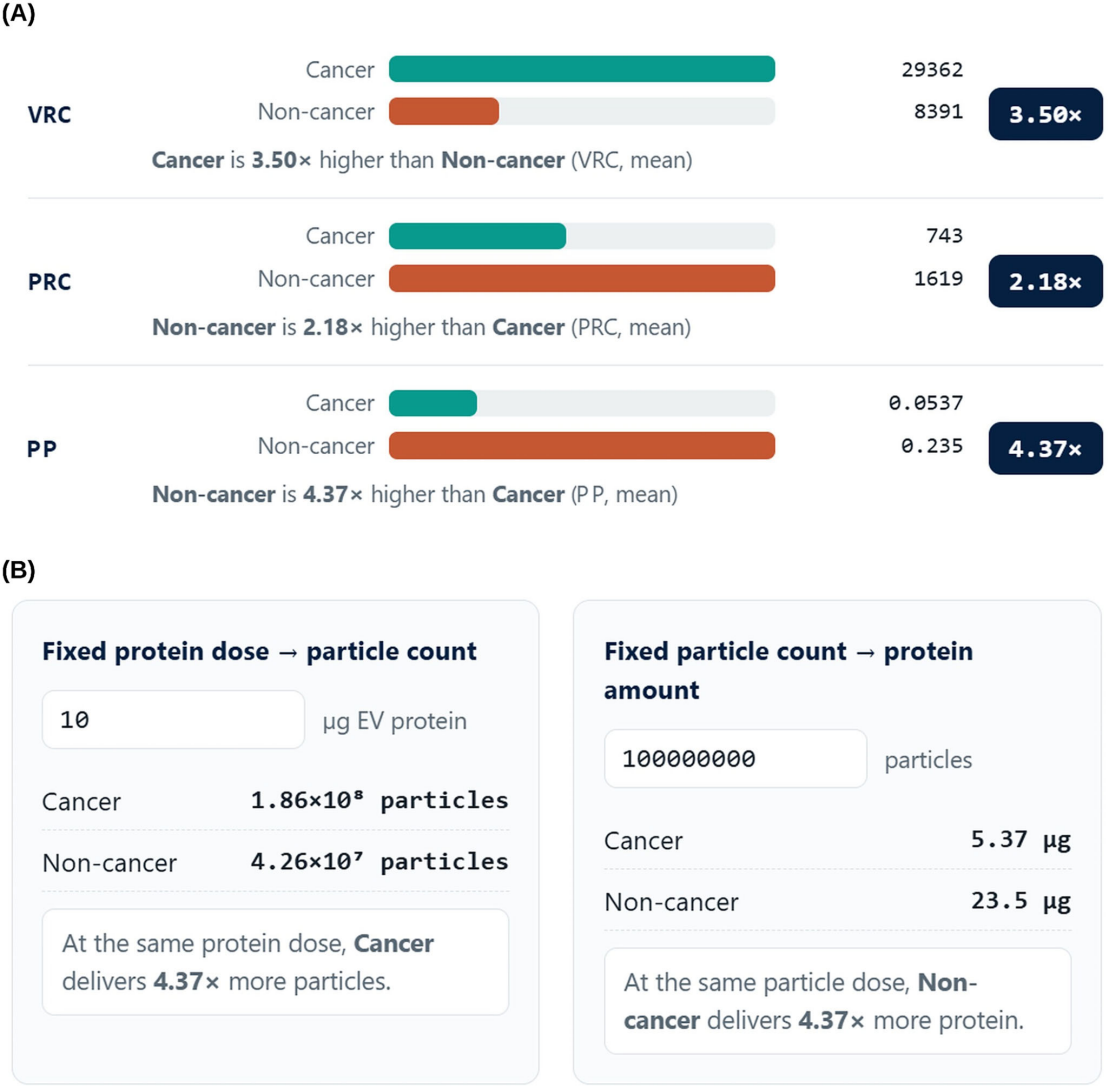
Web‐based calculation of particle‐ and protein‐related metrics and dose normalization. (A) Group mean VRC, PRC and PP values with the corresponding fold differences between cancer and non‐cancer cell lines. (B) Bidirectional conversion between protein‐ and particle‐based doses of EV‐enriched isolates using the group‐specific mean PP values.

To support implementation, we developed a freely accessible web application that calculates the required metrics from the underlying measurements and directly converts between particle‐based and protein‐based doses of EV‐enriched isolates (Figure 7A–B). The tool requires only the core input variables underlying the framework: initial and final cell number, incubation time, conditioned medium volume, particle concentration, and protein concentration. All calculations are performed locally in the user's browser, and no uploaded data are stored or transmitted.

The web‐based tool is available at the following link: https://matyasbukva.github.io/ev‐normalization‐tool/

## 4 Discussion

In many EV studies, vesicle quantity is operationally defined either by particle number or by total EV‐associated protein, implicitly assuming that a given protein mass corresponds to a proportional number of vesicles (and vice versa), largely independent of cellular origin (Gupta et al. 2021). However, multiple observations challenge this assumption: increased EV yield can coincide with a marked reduction in protein per vesicle—a ‘protein dilution effect’ described in bioreactor‐based systems (Kronstadt et al. 2023; Patel et al. 2019),—and similar decoupling has been reported under pharmacological perturbation, such as neutral sphingomyelinase inhibition increasing EV number while reducing protein per vesicle (Menck 2017). These findings have direct implications for dose definition and cross‐study comparability, and they align with the MISEV2023 emphasis on multidimensional reporting (Welsh et al. 2024).

Importantly, these methodological and conceptual issues are particularly relevant when comparing EVs from cancer and non‐cancer cells, as cancer cells often exhibit higher EV release. Functional comparisons between cancer‐derived and non‐cancer EVs are usually framed in terms of ‘more EVs’ and ‘altered cargo’ (Clancy and D'Souza‐Schorey 2023; Hánělová et al. 2024; Kalluri and McAndrews 2023; Najaflou et al. 2022), yet EV number and EV‐associated protein are rarely quantified within a single, integrated framework at the per‐vesicle and per‐cell level.

In light of this, the present work addresses this gap by mapping, within a unified measurement framework, the relationships between EV release rate, protein‐based secretion parameters, and doubling time and by determining how cancer‐derived origin shifts these characteristics relative to non‐cancer cell lines.

Our results highlight four closely interconnected patterns. (i) Cancer cell lines showed significantly higher VRC values (particles/cell/24 h); (ii) their EV‐enriched isolates exhibited significantly lower PP (pg protein/particle) values; (iii) they also showed lower PRC values, including at comparable VRC, while the relationship between VRC and PRC differed between cancer and non‐cancer cell lines; and (iv) proliferation showed a parallel pattern, with faster‐dividing cell lines exhibiting lower PP values.

Before these patterns are interpreted biologically, it is necessary to be explicit about what the underlying measurements resolve. EV quantity was defined here by two bulk readouts, neither of which is strictly EV‐specific: NTA counted any sufficiently light‐scattering particle with a Brownian motion within its detection range, including lipoproteins and protein aggregates, and the BCA assay quantifies total protein irrespective of whether it is enclosed in a vesicle, membrane‐associated, adsorbed, or co‐isolated from the conditioned medium.

Our data support that particle number and recovered protein are not interchangeable descriptors, and that the factor converting one into the other differs systematically with cellular source—with concordant across two isolation workflows, independently of particle size and of the species composition of the panel, and after adjustment for proliferation. Because these are precisely the quantities used in practice to define EV dose, the resulting mismatch matters irrespective of how much of the measured protein is vesicular. What we can say more cautiously is that our data do not, on their own, establish that individual cancer‐derived vesicles carry less protein. Two non‐biological explanations remain open and cannot yet be ruled out: PP is, by construction, protein divided by particle number, so a group difference in particle count alone could produce an inverse difference in this ratio; and cancer and non‐cancer cultures may release differing amounts of non‐vesicular protein or non‐vesicular particles into the medium. Given these open possibilities, we now treat the biological interpretation of PP as a hypothesis to be tested further, rather than as an established result.

As a **first key finding**, cancer‐derived cell lines showed higher estimated EV release (VRC), in line with previous reports. More broadly, enhanced EV release by cancer cells is one of the most robust observations in the field. Classic studies already suggested that cancer‐derived vesicles (exosomes/microvesicles) are active components of the communication and adaptation toolkit of cancer cells (e.g., growth‐supporting protein and RNA cargo in glioblastoma microvesicles) (Skog et al. 2008). Direct NTA‐based counting has consistently reported higher particle yields from cancer cells (Ahn et al. 2025; Balaj et al. 2011).

Mechanistic studies of cancer‐derived EVs have identified multiple cancer‐associated pathways that drive EV production upward, including Rab27a/b‐mediated exosome secretion (Ostrowski et al. 2010), increased exosome release associated with invadopodia under invasive phenotypes (Hoshino et al. 2013), and oncogenic RAS‐induced increased EV emission (Lee et al. 2014).

In this context, our observation that cancer‐derived cell lines exhibit higher VRC fits well into this literature: cancer cell EV release is not a mere byproduct but a regulated output that varies together with cell biological programs linked to cancer progression.

As a **second key finding**, higher VRC (particles/cell/24 h) in cancer‐derived cell lines was accompanied by lower PP (pg protein/particle) and was not matched by higher PRC (pg protein/cell/24 h). This quantitative imbalance raises the question of which cancer‐associated pathways support such high estimated EV release.

Regulation of EV secretion can also be interpreted through several cancer‐associated signalling axes. Beyond the pathways discussed above, EV secretion can also be influenced by stress‐responsive genetic programs. The p53‐linked TSAP6/Steap3 axis, for example, indicates that in DNA damage– and stress‐related contexts, exosome secretion can come under direct genetic control (Lespagnol et al. 2008). Moreover, cancer cells are exposed to cellular stress and microenvironmental constraints, such as hypoxia, nutrient limitation, oxidative stress, all of which can modulate EV release. Under hypoxia, cancer cells can increase exosome/EV secretion and shift towards a pro‐angiogenic vesicular program (e.g., glioma and breast cancer models) (King et al. 2012; Kucharzewska et al. 2013). In addition to endogenous processes, exogenous influences such as chemotherapy can also modulate vesicle release by cancer cells (Harmati et al. 2019).

In this context—as our **third key finding**—our results demonstrate that cancer‐origin cell lines show lower PRC (pg protein/cell/24 h) than non‐cancer cells, even at comparable levels of VRC (particles/cell/24 h). Moreover, while non‐cancer cells show an inverse relationship between VRC and PRC (higher VRC coincides with lower PRC), cancer cells show a different VRC–PRC relationship, in which higher VRC is not accompanied by the same decrease in PRC. This indicates that the ‘protein dilution effect’, defined here at the level of particle and protein measurements in EV‐enriched isolates, is detectable in non‐cancer cell lines in our dataset but is not observed in the same form in cancer‐derived cells.

This biological picture aligns with the broader view that the cancer EV secretory strategy is not to ‘export more protein’ but to redistribute output—reaching more target cells across more microenvironmental niches to re‐educate the tumour microenvironment and build pro‐invasive, pro‐angiogenic, immunomodulatory networks, even with smaller or specialized cargo per vesicle (Kalluri and LeBleu 2020).

Taken together, these mechanisms support the view that secretion in cancer cells is ‘less constrained’ and can operate over a broader dynamic range.

In our dataset, proliferation emerges as a quantitative axis rather than a mere background variable, co‐varying with the particle–protein characteristics of EV‐enriched isolates. The negative association between proliferation rate and PP (pg protein/particle) suggests that rapid growth programs are associated not only with increased cell division but also with lower protein‐to‐particle ratios in EV‐enriched isolates.

This could be explained by a number of biological processes. Proliferating cells reprogram membrane trafficking and the endosomal system, and EV release has been proposed to occur in a cell cycle phase–dependent manner. Recent cell cycle–focused data suggest that structures linked to mitosis/cytokinesis (e.g., midbody remnants) may intersect with EV release across different size ranges, and that inhibition of proliferation reduces multiple EV fractions (Patel et al. 2024).

Importantly, our multivariable analyses indicate that cancer status and proliferation jointly explain a substantial fraction of the variance in PP (pg protein/particle), and the association with cancer status remains after adjustment for proliferation. This implies that cancer origin is not simply a proxy for faster growth but is associated with additional differences in the particle–protein characteristics of EV‐enriched isolates. Potential biological mechanisms underlying such differences are suggested by previous studies showing direct remodelling of EV release and composition by oncogenic RAS (Choi et al. 2021; Lee et al. 2014) and reprogramming of Rab27 and nSMase2/ceramide secretory nodes in cancer (Kosaka et al. 2013; Ostrowski et al. 2010). These data support treating proliferation as a covariate or stratification factor in comparative EV studies.

One of the most important messages of our findings for EV experimental design is the potential mismatch introduced by protein‐only or particle‐only normalization. However, MISEV guidelines already emphasize that total protein and particle number are both approximate quantitative descriptors, and neither can be considered a universally ‘correct’ dosing metric; parallel reporting of multiple quantitative metrics (particle number, protein, lipid, starting cell number/volume) is recommended (Welsh et al. 2024). The EV‐TRACK initiative similarly highlights the lack of transparent reporting as a reproducibility risk (EV‐TRACK Consortium 2017).

Our data make this methodological issue concrete in a simple biological comparison. Based on the mean PP (pg protein/particle) values, EV‐enriched isolates from cancer‐derived cell lines contained 4.37‐fold less protein per detected particle than those from non‐cancer cell lines. Consequently, matching isolates by protein alone would result in a 4.37‐fold difference in particle number, whereas matching them by particle number alone would result in a 4.37‐fold difference in protein amount in the opposite direction. Thus, functional comparisons based on only one of these normalization metrics may introduce substantial and systematic differences in the other, potentially complicating the interpretation of source‐dependent effects.

The particle‐to‐protein ratio is often discussed as a purity indicator (Webber and Clayton 2013). However, our results suggest that PP (pg protein/particle) may also vary systematically with biological source and therefore should not be interpreted solely as a measure of isolate purity. This has direct implications for EV dose normalization: when PP differs between sources, matching EV‐enriched isolates by protein amount alone will result in different particle numbers, whereas matching by particle number will result in different protein amounts. Accordingly, particle‐ and protein‐based measurements should be reported together rather than treated as interchangeable normalization metrics. In line with the practical framework introduced in Section 3.7, we therefore recommend reporting VRC (particles/cell/24 h), PRC (pg protein/cell/24 h), and PP (pg protein/particle) in studies assessing EV release and associated protein recovery. For functional experiments in which EV‐enriched isolates are applied to recipient cells, we recommend reporting both the applied particle number and protein amount, together with the corresponding PP (pg protein/particle) value. These considerations are particularly relevant to studies in which particle number and protein are quantified by NTA and BCA, respectively, as in the present study.

This consideration is not limited to functional dosing. It also applies to experiments requiring a defined input of EVs, including quantitative proteomics, ELISA, and immunoblotting. When PP differs between biological sources, normalization by protein amount or particle number results in different quantities of the complementary metric being analyzed, which may bias marker‐abundance estimates and biomarker comparisons.

With these broader implications in mind, our analysis itself is deliberately positioned at the level of bulk quantitative dosing metrics—BCA‐detectable protein and NTA‐detectable particle number in EV‐enriched isolates—rather than cargo composition. The decoupling we report holds irrespective of which proteins underlie the bulk difference, and total protein is commonly used as a basis for protein‐based dosing. We did not determine whether the lower PP observed in cancer‐derived EV‐enriched isolates reflects differences in vesicle‐associated protein, non‐vesicular protein, particle composition, or a combination of these factors; resolving this will require dedicated vesicle‐resolved and quantitative proteomic analyses and represents an important complementary direction. For the same reason, marker‐based signals such as tetraspanin abundance are not proposed here as a normalization axis, since tetraspanin expression itself varies by cell source and EV subpopulation and would be subject to source‐dependent variability.

Moreover, it is worth mentioning that the cancer‐derived panel was intentionally diverse in tissue origin and oncogenic background, and the resulting heterogeneity in vesicle output is expected. Proliferation rate and cancer origin account for systematic, reproducible components of this variation, but not for all line‐to‐line differences. Part of this residual heterogeneity may reflect line‐specific shifts in the balance between ESCRT‐dependent and ESCRT‐independent biogenesis pathways, both of which operate in parallel in non‐cancer cells but can become imbalanced in cancer cells through upregulation of specific components (e.g., Rab31 and nSMase2 favouring ESCRT‐independent, ceramide‐driven release; Tsg101 and ALIX favouring ESCRT‐dependent release) (Horbay et al. 2022; Lee et al. 2024; Xie et al. 2022). This may occur alongside the other candidate mechanisms discussed above (Rab27, invadopodia‐associated release, oncogenic RAS, p53–TSAP6, hypoxia/nSMase2) (Hoshino et al. 2013; Hoshino et al. 2013; Lee et al. 2014). Mapping the expression of these machineries onto per‐line EV output is an important mechanistic question beyond the scope of the present quantitative analysis.

Establishing whether the difference reported here is carried by the vesicles themselves requires vesicle‐resolved validation beyond the scope of the present work: fraction‐resolved measurement of particle number, protein and EV markers in parallel; detergent‐ and protease‐based experiments to determine what share of the counted particles is membrane‐enclosed and what share of the measured protein is vesicle‐protected, applied across representative cancer and non‐cancer lines rather than the single line characterized here; assessment of non‐EV contaminants by density separation; and orthogonal single‐particle counting to estimate the EV‐attributable particle fraction. Until such data are available, we deliberately restrict our conclusions to the level at which the measurements were made.

## Author Contributions

Conceptualization: **Tímea Böröczky**, **Mátyás Bukva** and **Mária Harmati**. Data curation: Tímea Böröczky. Formal analysis: Mátyás Bukva and **Antal Martinecz**. Funding acquisition: **Krisztina Buzás** and Tímea Böröczky. Investigation: Tímea Böröczky, **Tamás F. Polgár**, **Edina Gyukity‐Sebestyén** and **Csenge Feinek**. Methodology: Tímea Böröczky, Mária Harmati, Mátyás Bukva and **Gabriella Dobra**. Project administration: Gabriella Dobra. Resources: Krisztina Buzás. Supervision: Krisztina Buzás, Gabriella Dobra and Mária Harmati. Validation: Mária Harmati and Gabriella Dobra. Visualization: Tímea Böröczky, **Emma Balog** and Mátyás Bukva. Writing – original draft: Tímea Böröczky, Mátyás Bukva and Gabriella Dobra. Writing – review and editing: Tímea Böröczky, Gabriella Dobra and Mária Harmati

## Funding

The work was supported by the following research grants: OTKA‐K143255 (K.B.), TKP‐2021‐EGA‐09 (K.B.), and University Excellence Scholarship Program EKOP‐24‐3‐SZTE‐318 (T.B.). This work was carried out with the professional support of the Ministry of Culture and Innovation, funded by the National Research, Development and Innovation Fund.

## Conflicts of Interest

The authors declare that there is no conflict of interest.

## Supporting information




**Supporting Information**: jex270188‐sup‐0001‐FigureS1.docx

**Supporting Information**: jex270188‐sup‐0002‐FigureS2.docx

**Supporting Information**: jex270188‐sup‐0003‐FigureS3.docx

**Supporting Information**: jex270188‐sup‐0004‐TableS1.docx

**Supporting Information**: jex270188‐sup‐0005‐SuppMat.docx

## Data Availability

All datasets generated during the current study are available from https://github.com/matyasbukva/ev_quantitative. All relevant data of our experiments have been submitted to the EV‐TRACK (Van Deun J, et al. EV‐TRACK: transparent reporting and centralizing knowledge in extracellular vesicle research knowledgebase^33^ **(EV‐TRACK ID: EV260010)**. To make this multidimensional reporting scheme practical for other laboratories, we also developed a dedicated application to assist with the calculations: https://matyasbukva.github.io/ev‐normalization‐tool/.

## References

[jex270188-bib-0001] Ahn, M. , J.‐G. Mun , Y. Han , and J. H. Seo . 2025. “Cancer Cell‐Derived Extracellular Vesicles: A Potential Target for Overcoming Tumor Immunotherapy Resistance and Immune Evasion Strategies.” Frontiers in Immunology 16: 1601266. 40574844 10.3389/fimmu.2025.1601266 PMC12198139

[jex270188-bib-0002] Balaj, L. , R. Lessard , L. Dai , et al. 2011. “Tumour Microvesicles Contain Retrotransposon Elements and Amplified Oncogene Sequences.” Nature Communications 2: 180. 10.1038/ncomms1180 PMC3040683 21285958

[jex270188-bib-0003] Böröczky, T. 2023. “Impact of Experimental Conditions on Extracellular Vesicles' Proteome: A Comparative Study.” Life 13: 206. 36676155 10.3390/life13010206 PMC9864048

[jex270188-bib-0004] Casajuana Ester, M. , and R. M. Day . 2023. “Production and Utility of Extracellular Vesicles With 3D Culture Methods.” Pharmaceutics 15: 663. 36839984 10.3390/pharmaceutics15020663 PMC9961751

[jex270188-bib-0005] Chen, T.‐Y. , E. Gonzalez‐Kozlova , T. Soleymani , et al. 2022. “Extracellular Vesicles Carry Distinct Proteo‐Transcriptomic Signatures That Are Different From Their Cancer Cell of Origin.” Iscience 25: 104414. 35663013 10.1016/j.isci.2022.104414 PMC9157216

[jex270188-bib-0006] Choi, D. , L. Montermini , B. Meehan , A. Lazaris , P. Metrakos , and J. Rak . 2021. “Oncogenic RAS Drives the CRAF‐Dependent Extracellular Vesicle Uptake Mechanism Coupled With Metastasis.” Journal of Extracellular Vesicles 10: e12091. 34136107 10.1002/jev2.12091 PMC8191585

[jex270188-bib-0007] Choi, D.‐S. , D.‐Y. Choi , B. Hong , et al. 2012. “Quantitative Proteomics of Extracellular Vesicles Derived From human Primary and Metastatic Colorectal Cancer Cells.” Journal of Extracellular Vesicles 1: 18704. 10.3402/jev.v1i0.18704 PMC3760640 24009881

[jex270188-bib-0008] Clancy, J. W. , and C. D'Souza‐Schorey . 2023. “Tumor‐Derived Extracellular Vesicles: Multifunctional Entities in the Tumor Microenvironment.” Annual Review of Pathology: Mechanisms of Disease 18: 205–229. 10.1146/annurev-pathmechdis-031521-022116 PMC10410237 36202098

[jex270188-bib-0009] Costa‐Silva, B. , N. M. Aiello , A. J. Ocean , et al. 2015. “Pancreatic Cancer Exosomes Initiate Pre‐Metastatic Niche Formation in the Liver.” Nature Cell Biology 17: 816–826. 25985394 10.1038/ncb3169 PMC5769922

[jex270188-bib-0010] EV‐TRACK Consortium . 2017. “EV‐TRACK: Transparent Reporting and Centralizing Knowledge in Extracellular Vesicle Research.” Nature Methods 14: 228–232. 28245209 10.1038/nmeth.4185

[jex270188-bib-0011] Figueroa‐Valdés, A. I. , C. de la Fuente , Y. Hidalgo , et al. 2021. “A Chemically Defined, Xeno‐ and Blood‐Free Culture Medium Sustains Increased Production of Small Extracellular Vesicles from Mesenchymal Stem Cells.” Frontiers in Bioengineering and Biotechnology 9: 619930. 34124014 10.3389/fbioe.2021.619930 PMC8187876

[jex270188-bib-0012] Geng, T. , S. Y. Paek , E. Leung , L. W. Chamley , and Z. Wu . 2024. “Comparing Extracellular Vesicles From Four Different Cell Origins for Intracellular Drug Delivery to Pancreatic Cancer Cells: Small or Large Vesicles?” Journal of Drug Delivery Science and Technology 93: 105416.

[jex270188-bib-0013] Goudsmit, C. , F. da Veiga Leprevost , V. Basrur , L. Peters , A. Nesvizhskii , and H. Walline . 2021. “Differences in Extracellular Vesicle Protein Cargo Are Dependent on Head and Neck Squamous Cell Carcinoma Cell of Origin and Human Papillomavirus Status.” Cancers 13: 3714. 34359613 10.3390/cancers13153714 PMC8345072

[jex270188-bib-0014] Guerreiro, E. M. , R. Øvstebø , B. Thiede , D. E. Costea , T. M. Søland , and H. Kanli Galtung . 2020. “Cancer Cell Line‐Specific Protein Profiles in Extracellular Vesicles Identified by Proteomics.” PLoS ONE 15: e0238591. 32886718 10.1371/journal.pone.0238591 PMC7473518

[jex270188-bib-0015] Gupta, D. , A. M. Zickler , and S. El Andaloussi . 2021. “Dosing Extracellular Vesicles.” Advanced Drug Delivery Reviews 178: 113961. 34481030 10.1016/j.addr.2021.113961

[jex270188-bib-0016] Hánělová, K. , M. Raudenská , M. Masařík , and J. Balvan . 2024. “Protein Cargo in Extracellular Vesicles as the Key Mediator in the Progression of Cancer.” Cell Communication and Signaling 22: 25 . 38200509 10.1186/s12964-023-01408-6 PMC10777590

[jex270188-bib-0017] Harmati, M. , E. Gyukity‐Sebestyen , G. Dobra , et al. 2019. “Small Extracellular Vesicles Convey the Stress‐Induced Adaptive Responses of Melanoma Cells.” Scientific Reports 9: 15329. 31653931 10.1038/s41598-019-51778-6 PMC6814750

[jex270188-bib-0018] Horbay, R. , A. Hamraghani , L. Ermini , S. Holcik , S. T. Beug , and B. Yeganeh . 2022. “Role of Ceramides and Lysosomes in Extracellular Vesicle Biogenesis, Cargo Sorting and Release.” International Journal of Molecular Sciences 23, no. 23: 15317. 10.3390/ijms232315317. 36499644 PMC9735581

[jex270188-bib-0019] Hoshino, A. , B. Costa‐Silva , T.‐L. Shen , et al. 2015. “Tumour Exosome Integrins Determine Organotropic Metastasis.” Nature 527: 329–335. 26524530 10.1038/nature15756 PMC4788391

[jex270188-bib-0020] Hoshino, D. , K. C. Kirkbride , K. Costello , et al. 2013. “Exosome Secretion Is Enhanced by Invadopodia and Drives Invasive Behavior.” Cell Reports 5: 1159–1168. 24290760 10.1016/j.celrep.2013.10.050 PMC3873329

[jex270188-bib-0021] Huang, J. , H. Chen , N. Li , P. Liu , J. Yang , and Y. Zhao . 2025. “Emerging Technologies Towards Extracellular Vesicles Large‐Scale Production.” Bioactive Materials 52: 338–365. 40585384 10.1016/j.bioactmat.2025.06.005 PMC12206051

[jex270188-bib-0022] Hurwitz, S. N. , M. A. Rider , J. L. Bundy , X. Liu , R. K. Singh , and D. G. Meckes . 2016. “Proteomic Profiling of NCI‐60 Extracellular Vesicles Uncovers Common Protein Cargo and Cancer Type‐specific Biomarkers.” Oncotarget 7: 86999–87015. 27894104 10.18632/oncotarget.13569 PMC5341331

[jex270188-bib-0023] JASP Team . JASP (Version 0.95.4)[Computer software]. 2025.

[jex270188-bib-0024] Kalluri, R. , and V. S. LeBleu . 2020. “The Biology, Function, and Biomedical Applications of Exosomes.” Science 367: eaau6977. 32029601 10.1126/science.aau6977 PMC7717626

[jex270188-bib-0025] Kalluri, R. , and K. M. McAndrews . 2023. “The Role of Extracellular Vesicles in Cancer.” Cell 186: 1610–1626. 37059067 10.1016/j.cell.2023.03.010 PMC10484374

[jex270188-bib-0026] Kim, J. Y. , W.‐K. Rhim , H. J. Seo , J. Y. Lee , C. G. Park , and D. K. Han . 2021. “Comparative Analysis of MSC‐Derived Exosomes Depending on Cell Culture Media for Regenerative Bioactivity.” Tissue Engineering and Regenerative Medicine 18: 355–367. 34047999 10.1007/s13770-021-00352-1 PMC8169723

[jex270188-bib-0027] King, H. W. , M. Z. Michael , and J. M. Gleadle . 2012. “Hypoxic Enhancement of Exosome Release by Breast Cancer Cells.” BMC cancer 12: 421. 22998595 10.1186/1471-2407-12-421 PMC3488584

[jex270188-bib-0028] Kobayashi, H. , T. Shiba , T. Yoshida , et al. 2024. “Precise Analysis of Single Small Extracellular Vesicles Using Flow Cytometry.” Scientific Reports 14: 7465. 38553534 10.1038/s41598-024-57974-3 PMC10980769

[jex270188-bib-0029] Kosaka, N. , H. Iguchi , K. Hagiwara , Y. Yoshioka , F. Takeshita , and T. Ochiya . 2013. “Neutral Sphingomyelinase 2 (nSMase2)‐Dependent Exosomal Transfer of Angiogenic MicroRNAs Regulate Cancer Cell Metastasis.” Journal of Biological Chemistry 288: 10849–10859. 23439645 10.1074/jbc.M112.446831 PMC3624465

[jex270188-bib-0030] Kronstadt, S. M. , D. B. Patel , L. J. Born , et al. 2023. “Mesenchymal Stem Cell Culture Within Perfusion Bioreactors Incorporating 3D‐Printed Scaffolds Enables Improved Extracellular Vesicle Yield With Preserved Bioactivity.” Advanced Healthcare Materials 12: 2300584. 10.1002/adhm.202300584 PMC10505252 36930747

[jex270188-bib-0031] Kucharzewska, P. , H. C. Christianson , J. E. Welch , et al. 2013. “Exosomes Reflect the Hypoxic Status of Glioma Cells and Mediate Hypoxia‐Dependent Activation of Vascular Cells During Tumor Development.” Proceedings of the National Academy of Sciences 110: 7312–7317. 10.1073/pnas.1220998110 PMC3645587 23589885

[jex270188-bib-0032] Lee, T. H. , S. Chennakrishnaiah , E. Audemard , L. Montermini , B. Meehan , and J. Rak . 2014. “Oncogenic Ras‐Driven Cancer Cell Vesiculation Leads to Emission of Double‐Stranded DNA Capable of Interacting With Target Cells.” Biochemical and Biophysical Research Communications 451: 295–301. 25086355 10.1016/j.bbrc.2014.07.109

[jex270188-bib-0033] Lee, Y. J. , K. J. Shin , and Y. C. Chae . 2024. “Regulation of Cargo Selection in Exosome Biogenesis and Its Biomedical Applications in Cancer.” Experimental & Molecular Medicine 56, no. 4: 877–889. 10.1038/s12276-024-01209-y. 38580812 PMC11059157

[jex270188-bib-0034] Lespagnol, A. , D. Duflaut , C. Beekman , et al. 2008. “Exosome Secretion, Including the DNA Damage‐Induced p53‐Dependent Secretory Pathway, Is Severely Compromised in TSAP6/Steap3‐Null Mice.” Cell Death & Differentiation 15: 1723–1733. 18617898 10.1038/cdd.2008.104

[jex270188-bib-0035] Lopez, K. , S. W. T. Lai , E. D. J. Lopez Gonzalez , R. G. Dávila , and S. C. Shuck . 2023. “Extracellular Vesicles: A Dive Into Their Role in the Tumor Microenvironment and Cancer Progression.” Frontiers in Cell and Developmental Biology 11: 1154576. 37025182 10.3389/fcell.2023.1154576 PMC10071009

[jex270188-bib-0036] Ma, S. , J. Ene , C. McGarraugh , et al. 2025. “Extracellular Vesicle Secretion From 3D Culture of Human Adipose‐Derived Mesenchymal Stem Cells in Scalable Bioreactors.” Bioengineering 12: 933. 41007177 10.3390/bioengineering12090933 PMC12467516

[jex270188-bib-0037] Menck, K. , C. Sönmezer , T. S. Worst , et al. 2017. “Neutral Sphingomyelinases Control Extracellular Vesicles Budding From the Plasma Membrane.” Journal of Extracellular Vesicles 6: 1378056. 29184623 10.1080/20013078.2017.1378056 PMC5699186

[jex270188-bib-0038] Najaflou, M. , M. Shahgolzari , A. Y. Khosroushahi , and S. Fiering . 2022. “Tumor‐Derived Extracellular Vesicles in Cancer Immunoediting and Their Potential as Oncoimmunotherapeutics.” Cancers 15: 82. 36612080 10.3390/cancers15010082 PMC9817790

[jex270188-bib-0039] Ostrowski, M. , N. B. Carmo , S. Krumeich , et al. 2010. “Rab27a and Rab27b Control Different Steps of the Exosome Secretion Pathway.” Nature Cell Biology 12: 19–30. 19966785 10.1038/ncb2000

[jex270188-bib-0040] Patel, D. B. , C. R. Luthers , M. J. Lerman , J. P. Fisher , and S. M. Jay . 2019. “Enhanced Extracellular Vesicle Production and Ethanol‐Mediated Vascularization Bioactivity Via a 3D‐Printed Scaffold‐Perfusion Bioreactor System.” Acta Biomaterialia 95: 236–244. 30471476 10.1016/j.actbio.2018.11.024 PMC6531369

[jex270188-bib-0041] Patel, S. A. , S. Park , D. Zhu , et al. 2024. “Extracellular Vesicles, Including Large Translating Vesicles Called Midbody Remnants, Are Released During the Cell Cycle.” Molecular Biology of the Cell 35: ar155. 39535882 10.1091/mbc.E23-10-0384 PMC11656471

[jex270188-bib-0042] Pei, H. , H. Si , D. Du , L. Li , and B. Tang . 2025. “Time‐Resolved Quantification of Single‐Cell Extracellular Vesicles Secretion for Drug Resistance Evaluation.” Analytical Chemistry 97: 15753–15761. 40658044 10.1021/acs.analchem.5c01558

[jex270188-bib-0043] Peinado, H. , M. Alečković , S. Lavotshkin , et al. 2012. “Melanoma Exosomes Educate Bone Marrow Progenitor Cells Toward a Pro‐Metastatic Phenotype Through MET.” Nature Medicine 18: 883–891. 10.1038/nm.2753 PMC3645291 22635005

[jex270188-bib-0044] Poggio, M. , T. Hu , C.‐C. Pai , et al. 2019. “Suppression of Exosomal PD‐L1 Induces Systemic Anti‐Tumor Immunity and Memory.” Cell 177: 414–427.e13. 30951669 10.1016/j.cell.2019.02.016 PMC6499401

[jex270188-bib-0045] Priya, R. , V. Jain , J. Akhtar , et al. 2022. “Proteomic Profiling of Cell Line‐Derived Extracellular Vesicles to Identify Candidate Circulatory Markers for Detection of Gallbladder Cancer.” Frontiers in Oncology 12: 1027914. 36505879 10.3389/fonc.2022.1027914 PMC9727277

[jex270188-bib-0046] Semeradtova, A. , M. Liegertova , R. Herma , M. Capkova , C. Brignole , and G. Del Zotto . 2025. “Extracellular Vesicles in Cancer´s Communication: Messages We Can Read and How to Answer.” Molecular Cancer 24: 86. 40108630 10.1186/s12943-025-02282-1 PMC11921637

[jex270188-bib-0047] Servage, K. A. , K. Stefanius , H. F. Gray , and K. Orth . 2020. “Proteomic Profiling of Small Extracellular Vesicles Secreted by Human Pancreatic Cancer Cells Implicated in Cellular Transformation.” Scientific Reports 10: 7713. 32382024 10.1038/s41598-020-64718-6 PMC7205864

[jex270188-bib-0048] Skog, J. , T. Würdinger , S. van Rijn , et al. 2008. “Glioblastoma Microvesicles Transport RNA and Proteins That Promote Tumour Growth and Provide Diagnostic Biomarkers.” Nature Cell Biology 10: 1470–1476. 19011622 10.1038/ncb1800 PMC3423894

[jex270188-bib-0049] Tabak, S. , S. Schreiber‐Avissar , and E. Beit‐Yannai . 2018. “Extracellular Vesicles Have Variable Dose‐Dependent Effects on Cultured Draining Cells in the Eye.” Journal of Cellular and Molecular Medicine 22: 1992–2000. 29411534 10.1111/jcmm.13505 PMC5824413

[jex270188-bib-0050] Webber, J. , and A. Clayton . 2013. “How Pure Are Your Vesicles?” Journal of Extracellular Vesicles 2: 19861. 10.3402/jev.v2i0.19861 PMC3760653 24009896

[jex270188-bib-0051] Welsh, J. A. , D. C. I. Goberdhan , L. O'Driscoll , et al. 2024. “Minimal Information for Studies of Extracellular Vesicles (MISEV2023): From Basic to Advanced Approaches.” Journal of Extracellular Vesicles 13: e12404. 38326288 10.1002/jev2.12404 PMC10850029

[jex270188-bib-0052] Welsh, J. A. , E. Van Der Pol , G. J. A. Arkesteijn , et al. 2020. “MIFlowCyt‐EV: A Framework for Standardized Reporting of Extracellular Vesicle Flow Cytometry Experiments.” Journal of Extracellular Vesicles 9: 1713526. 32128070 10.1080/20013078.2020.1713526 PMC7034442

[jex270188-bib-0053] Xie, S. , Q. Zhang , and L. Jiang . 2022. “Current Knowledge on Exosome Biogenesis, Cargo‐Sorting Mechanism and Therapeutic Implications.” Membranes 12, no. 5: 498. 10.3390/membranes12050498. 35629824 PMC9144303

[jex270188-bib-0054] Zhang, L. , Q. Lei , H. Wang , et al. 2019. “Tumor‐Derived Extracellular Vesicles Inhibit Osteogenesis and Exacerbate Myeloma Bone Disease.” Theranostics 9: 196–209. 30662562 10.7150/thno.27550 PMC6332790

