# Supplementary material for: When EV Dose Is Not What It Seems: Quantitative Mismatch Between Particle Number‐ and Protein‐Based Dosing
Source: J Extracell Biol. 2026 Sep 13;5(9):e70188. doi: 10.1002/jex2.70188 (PMC13572909; doi:10.1002/jex2.70188)
Supplement: Supplementary file 1 [file JEX2-5-e70188-s001.docx]

**Supplementary Figure 3**. Comparison of proliferation-associated EV protein relationships between SEC and dUC isolation. Scatter plots with linear regression lines and 95% confidence bands show the relationships Proliferation rate and VP or PRC in the six cell lines processed in parallel by SEC and dUC. Each point represents a single cell line. Pearson’s correlation coefficient (r) and the corresponding p-value are shown in each panel.
