# Supplementary material for: When EV Dose Is Not What It Seems: Quantitative Mismatch Between Particle Number‐ and Protein‐Based Dosing
Source: J Extracell Biol. 2026 Sep 13;5(9):e70188. doi: 10.1002/jex2.70188 (PMC13572909; doi:10.1002/jex2.70188)
Supplement: Supplementary file 2 [file JEX2-5-e70188-s002.docx]

**Supplementary Table 1**. The composition of the cell culture media

| **Cell culture** | **Cell type** | **Origin** | **Cell type** | **Culture medium** | **Supplements** |
| --- | --- | --- | --- | --- | --- |
| MRC-5 | fibroblast | human | non-cancer | EMEM | 1% MEM NEAA, 1% L-Glutamine |
| MSC | mesenchymal stem cell | mouse | non-cancer | MSC expansion media | MSC Supplement |
| Vero E6 | kidney epithelial | monkey | non-cancer | DMEM (4,5 g/L glucose) | 1% L-Glutamine |
| HOB-C | osteoblast | human | non-cancer | Human Osteoblast Growth Medium | 1% MEM NEAA; 1% MEM Vitamin; 1% L-Glutamine |
| MDCK | kidney epithelial | canine | non-cancer | DMEM (4,5 g/L glucose) | 1% L-Glutamine |
| Hek-293 | embryonic kidney | human | non-cancer | DMEM (4,5 g/L glucose) | - |
| 4T1 | mammary carcinoma | mouse | cancer | RPMI | - |
| T-47D | ductal carcinoma | human | cancer | RPMI | 0.2 unit/mL insulin |
| B16F1 | melanoma with low metastatic potential | mouse | cancer | DMEM (4,5 g/L glucose) | 1% MEM NEAA; 1% MEM vitamin, 0.01% Na-pyruvate; 1% L-Glutamine |
| B16F0 | melanoma | mouse | cancer | DMEM (4,5 g/L glucose) | 1% L-Glutamine |
| B16F10 | melanoma with high metastatic potential | mouse | cancer | DMEM (4,5 g/L glucose) | 1% L-Glutamine |
| HCT-116 | colorectal carcinoma | human | cancer | DMEM (4,5 g/L glucose) | 1% L-Glutamine |
| Huh-7D12 | hepatocellular carcinoma | human | cancer | DMEM (4,5 g/L glucose) | 1% L-Glutamine |
| PC-3 | prostate adenocarcinoma | human | cancer | DMEM F-12K | - |
| MG-63 | osteosarcoma | human | cancer | EMEM | 1% MEM NEAA; 1% Na-pyruvate; 1% L-Glutamine |
| Raw 264.7 | macrophages | mouse | cancer | DMEM F-12K | - |
| Jurkat | T lymphoblast | human | cancer | RPMI | 0. 1% mEtOH |
| Ramos | Burkitt lymphoma | human | cancer | RPMI | - |
| THP-1 | acute monocytic leukemia | human | cancer | RPMI | 0. 1% mEtOH |
|  |  |  |  |  |  |
