# Supplementary material for: When EV Dose Is Not What It Seems: Quantitative Mismatch Between Particle Number‐ and Protein‐Based Dosing
Source: J Extracell Biol. 2026 Sep 13;5(9):e70188. doi: 10.1002/jex2.70188 (PMC13572909; doi:10.1002/jex2.70188)
Supplement: Supplementary file 3 [file JEX2-5-e70188-s003.docx]

**Supplementary Figure 2**. Comparison of EV-related parameters between non-cancer and cancer cell lines using SEC and dUC isolation. VP, VRC, and PRC were compared in the same six cell lines processed in parallel by size-exclusion chromatography (SEC) and differential ultracentrifugation (dUC). Bars show generalized linear model-derived estimated means for non-cancer and cancer cell lines, and error bars indicate the corresponding 95% confidence intervals. Individual points represent single cell lines, with labels indicating cell-line identity. Blue indicates non-cancer cell lines and orange indicates cancer cell lines. The Cancer / Non-cancer fold-change and corresponding 95% confidence interval are shown in each panel.
