# Supplementary material for: When EV Dose Is Not What It Seems: Quantitative Mismatch Between Particle Number‐ and Protein‐Based Dosing
Source: J Extracell Biol. 2026 Sep 13;5(9):e70188. doi: 10.1002/jex2.70188 (PMC13572909; doi:10.1002/jex2.70188)
Supplement: Supplementary file 4 [file JEX2-5-e70188-s004.docx]

**Supplementary Figure 1.** Representative TEM images of dUC and SEC-isolated EVs from MDCK, THP-1, VERO and RAMOS cell culture supernatant. Representative transmission electron microscopy images showing extracellular vesicle preparations isolated from cell culture supernatant by differential centrifugation (dUC) (panel A, B, C) and size-exclusion chromatography (SEC) (panel D, E).
