# Supplementary material for: When EV Dose Is Not What It Seems: Quantitative Mismatch Between Particle Number‐ and Protein‐Based Dosing
Source: J Extracell Biol. 2026 Sep 13;5(9):e70188. doi: 10.1002/jex2.70188 (PMC13572909; doi:10.1002/jex2.70188)
Supplement: Supplementary file 5 [file JEX2-5-e70188-s005.docx]

Supplementary Document 1:

Flow cytometry gating strategies for MACSPlex and bead-free Calcein AM analyses

**Associated manuscript**

*When EV Dose Is Not What It Seems: Quantitative Mismatch Between Particle Number- and Protein-Based Dosing*

# File organization

This file is organized into two sections. The first section contains representative MACSPlex flow cytometry plots presented in the following order: (1) capture beads, (2) PBS buffer control, (3) MRC-5 culture medium control, and (4) MRC-5 EV sample. The second section describes the particle-gating strategy used for the bead-free Calcein AM assay.

Each page uses the same four-panel arrangement. Upper left: FSC-A/SSC-A plot showing the P1 capture bead gate. Upper right: APC-A/PE-A plot showing the APC-positive gate. Lower left: FITC-A/PE-A plot showing the marker-specific bead populations. Lower right: event counts and median APC-A fluorescence values.

# 1. MACSPlex gating and quantification

These panels illustrate the gating approach applied during the analysis. The P1 gate was defined on the FSC/SSC plot to identify the capture bead population. The PBS control was then used to establish the APC-positive fluorescence threshold. Marker-specific bead populations within the APC-positive gate were subsequently identified and quantified. The median APC fluorescence intensity (MFI) of each capture bead population was used for statistical analysis and is summarized in Figure 1C.

# 2. Bead-free Calcein AM assay gating

For the bead-free Calcein AM assay, particle gating was performed using ApogeeMix sizing beads. Silica bead populations were used as reference particles, because their refractive index more closely resembles that of biological vesicles than that of polystyrene beads. The bead populations are explicitly annotated in the supplementary figure, indicating which reference beads are polystyrene (PS) and which are silica (SI). Based on these measurements, the P3 gate was defined below the 300 nm silica bead population. This gate was selected to capture small vesicle-sized particles while excluding background noise and the smallest detectable events. Representative ApogeeMix gating plots are provided in the Supplementary Materials to improve transparency and reproducibility.

# 1. Capture beads

Capture bead control showing the P1 bead population and the marker-specific bead subsets.

# 2. PBS buffer control

PBS buffer control used to establish the APC-positive fluorescence threshold.

# 3. MRC-5 culture medium control

MRC-5 culture medium control showing the background signal in the absence of isolated EVs.

# 4. MRC-5 EV sample

Representative MRC-5 EV sample showing APC-positive marker-specific bead populations used for MFI quantification.
